# An in‐depth Study of the Solid Electrolyte Interphase Compositional Evolution in Sodium‐Ion Batteries: Unravelling the Effects of a Na Metal Counter Electrode on the SEI

**DOI:** 10.1002/advs.202504717

**Published:** 2025-06-23

**Authors:** Jack R. Fitzpatrick, Beth E. Murdock, Pardeep K. Thakur, Tien‐Lin Lee, Sarah Fearn, Andrew J. Naylor, Deepnarayan Biswas, Nuria Tapia‐Ruiz

**Affiliations:** ^1^ Department of Chemistry Molecular Sciences Research Hub Imperial College London White City Campus London W12 0BZ UK; ^2^ The Faraday Institution Quad One Harwell Science and Innovation Campus Didcot OX11 0RA UK; ^3^ Diamond Light Source Ltd Harwell Science and Innovation Campus Didcot Oxfordshire OX11 0DE UK; ^4^ Department of Materials Imperial College London Exhibition Road London SW7 2AZ UK; ^5^ Department of Chemistry Ångström Laboratory Uppsala University Box 538 Uppsala 751 21 Sweden

**Keywords:** hard carbon, sodium‐ion batteries, solid electrolyte interphase, X‐ray photoelectron spectroscopy

## Abstract

A comprehensive understanding of the solid electrolyte interphase (SEI) is crucial for ensuring long‐term battery stability. This is particularly pertinent in sodium‐ion batteries (NIBs), where the SEI remains poorly understood, and investigations are typically undertaken in half‐cell configurations with sodium metal as the counter electrode. Na metal is known to be highly reactive with common carbonate‐based electrolytes; nevertheless, its effects on SEI formation at the working electrode are largely unexplored. This work investigates the evolution of the SEI in NIBs during cycling, with an emphasis on the consequences of using a sodium metal counter electrode. Advanced analytical techniques, including hard X‐ray photoelectron spectroscopy (HAXPES) and time‐of‐flight secondary ion mass spectrometry (ToF‐SIMS), are used to obtain depth‐resolved insights into the chemical composition and structural changes of the SEI on hard carbon anodes during cycling. The findings demonstrate that the cell configuration has a significant impact on SEI evolution and, by extension, battery performance. These findings suggest that full‐cell studies are necessary to better simulate practical operating conditions, challenging traditional half‐cell experiments.

## Graphical abstract

Compositional evolution of the solid electrolyte interphase in Na‐ion batteries is investigated, focusing on the effects of sodium metal counter electrodes on interphase formation. Advanced analytical techniques are employed to uncover distinct differences in interphase chemistry in half‐cell versus full‐cell configurations. These findings challenge traditional half‐cell approaches and underscore the need for full‐cell studies to optimize Na‐ion battery interfaces.

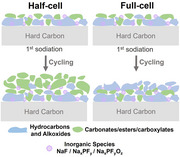

## 1 Introduction

Sodium‐ion batteries (NIBs) are rapidly approaching commercial viability, but our understanding of their solid electrolyte interphase (SEI) remains limited. Similar to lithium‐ion batteries (LIBs), the SEI in NIBs has a substantial impact on the long‐term electrochemical performance of Na‐based cells. The differing physicochemical features of Na^+^ and Li^+^ ions have a significant impact on their respective SEI layers.^[^ ^1^ ^]^ The SEI formed in Na‐ion‐based electrolytes is frequently thought to be less stable than its lithium‐based counterpart due to the increased solubility of the NIB SEI components.^[^ ^2^, ^3^ ^]^ The current understanding of the SEI in LIBs and NIBs is mostly based on half‐cell research, with Li and Na metals as counter electrodes (CE). In common carbonate‐based electrolytes used in battery research, Na metal is significantly more reactive than Li.^[^ ^2^, ^4^, ^5^, ^6^ ^]^ Studies on Na/Na symmetric cells have shown that Na metal initiates electrolyte degradation even at open circuit voltage, and the SEI composition of Na metal changes throughout cycling. This results in a considerable increase in overall interfacial resistance when compared to Li systems, affecting the long‐term cycling stability and rate capability of NIB half‐cells. The increased reactivity of Na metal also affects the composition, thickness, and stability of the SEI formed on the working electrode (WE). Electrolyte breakdown products at the Na metal surface have been shown to migrate through the separator and deposit onto the working electrode surface, affecting long‐term cycling stability.^[^ ^4^, ^5^, ^6^ ^]^ These considerations hinder the determination of the electrochemical characteristics of the working electrode in half‐cells.^[^ ^7^, ^8^ ^]^

Furthermore, the use of electrolyte additives in full‐cells warrants further exploration. Notably, the addition of fluoroethylene carbonate (FEC) has been demonstrated to improve the long‐term performance of NIB half‐cells. This improvement has been attributed to the formation of an SEI rich in both NaF and (sodium) alkyl carbonates on the hard carbon anode.^[^ ^9^, ^10^, ^11^ ^]^ However, the observed benefits of FEC addition are due in part to the surface passivation of the Na metal CE, which reduces sodium metal reactivity with the electrolyte, thereby preventing ongoing electrolyte decomposition and subsequent deposition of these species on the working electrode.^[^ ^6^, ^12^, ^13^ ^]^ Consequently, the use of Na metal as a CE has a significant impact on the overall cell electrochemistry, including SEI formation and composition at the working electrode.

This study uses hard X‐ray photoelectron spectroscopy (HAXPES) and time‐of‐flight secondary ion mass spectrometry (ToF‐SIMS) to compare depth‐resolved data and determine the compositional evolution of the SEI formed on hard carbon anodes in half‐cells (with Na metal CE) and three‐electrode full‐cells with a layered sodium transition metal oxide cathode. The study highlights the importance of cell setup selection in future NIB interface studies, particularly when using common carbonate‐based electrolytes with clear reactivity to sodium metal. Failure to consider this factor may result in incorrect conclusions about the fundamental understanding of SEI formation, evolution, and subsequent stability in NIBs, regardless of the chosen anode material used.

## 2 Results and Discussion

### 2.1 Electrochemical Testing In Half and Full‐Cells

**Figure 1:**
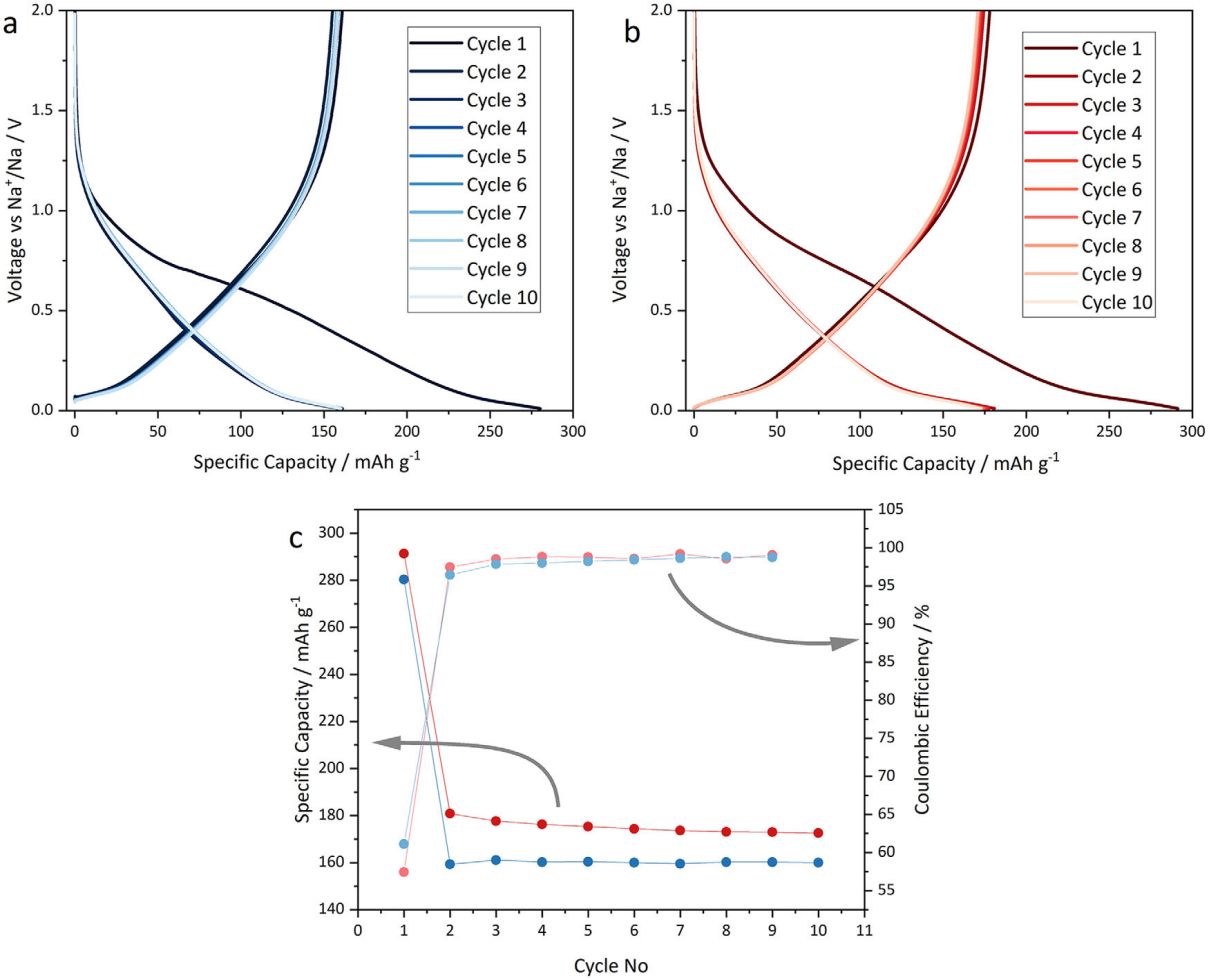
‐ Galvanostatic cycling data of the B187 hard carbon in a half‐ and full‐cell using a current of 15 mA g^−1^. Electrochemical data from a) a half‐cell with B187 as the negative electrode (anode) and Na metal as CE and b) a full‐cell with B187 as the anode and Na_0.79±0.05_Ni_0.27±0.05_Mn_0.42±0.05_Mg_0.15±0.05_Ti_0.17±0.05_O_2±0.05_ as the positive electrode (cathode). c) Specific capacity upon sodiation of the hard carbon and coulombic efficiency values vs. cycle number over the first 10 cycles. All the specific capacity values are plotted with respect to the hard carbon active material mass.

The hard carbon negative electrode used in this study (B187) was carbonized at 700 °C and has been previously characterized.^[^ ^14^ ^]^ This carbonization temperature is regarded as low compared to the carbonization temperatures commonly used in the literature.^[^ ^15^, ^16^, ^17^, ^18^, ^19^, ^20^, ^21^, ^22^ ^]^ This material was specifically chosen for this investigation due to its large surface area (326.8 m^2^ g^−1^), which promotes greater SEI formation and allows for more effective probing of its chemical composition. **Figure** **1** shows the electrochemical performance of this material in the half and full‐cell configurations used in this study during the first 10 cycles (see Figure S1 for schematics of the half and full‐cell configurations used). The voltage profiles are characteristic of a hard carbon material carbonized at ≲ 1000 °C, with a sloping region at V > 0.1 V and a minor plateau region at V < 0.1 V vs. Na^+^/Na.^[^ ^17^, ^18^, ^19^, ^20^ ^]^ Initial discharge (charge) capacities of 280 mAh g^−1^ (161 mAh g^−1^) and 291 mAh g^−1^ (178 mAh g^−1^) were achieved in the half‐cell and full‐cell configurations, respectively, with initial coulombic efficiencies (ICE) of 57 % and 61 %. After 10 cycles, there was only a slight difference in the specific capacity between the two cell types. Note S1.1 of the Supporting Information provides additional information about cell configurations.

### 2.2 HAXPES Data Analysis on Pristine Hard Carbon Electrode

**Figure 2:**
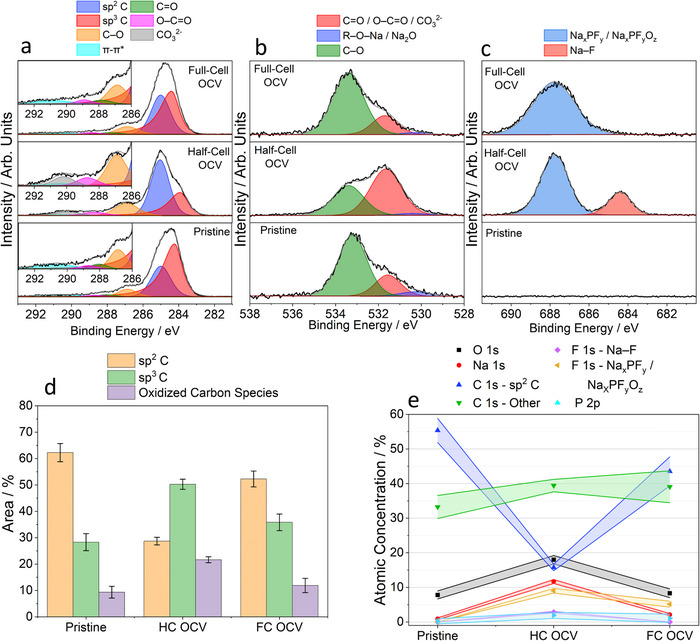
Ex situ HAXPES data obtained at 2350 eV. a) C 1s, b) O 1s, and c) F 1s spectra for hard carbon electrodes in the pristine state and extracted from half‐cells (HC) and full‐cells (FC) following a 10 h OCV period. d) Area percentage contributions of the sp^2^ C, sp^3^ C, and oxidized carbon species (C–O, C═O, O–C═O, and CO_3_^2−^) components to the C 1s spectra fittings. e) Calculated atomic concentrations of the elements/compounds (in at. %) and associated errors observed on the surface of the hard carbon electrodes in the pristine state and extracted from half‐cells and full‐cells after a 10 h OCV rest period. The plotted errors represent ± 2σ, where σ is the standard deviation in the fitted peak areas/atomic concentration calculated from the set of Monte Carlo simulations (refer to Note S1.2 in the Supporting Information).

Figures S2 and S3 show the survey spectra at 2350 and 7050 eV of the hard carbon negative electrode in the pristine state. At both energies, electrodes contain carbon, oxygen, and sodium‐containing species, as evidenced by peaks in the C 1s, O 1s, Na 1s, and Na 2s regions. As expected, the C 1s region has the highest peak intensity at both energies. **Figure** **2**a shows the C 1s spectrum of the pristine electrode. Two dominant peaks at 284.2 and 285.0 eV were observed, which correspond to sp^2^ C and sp^3^ C bonding, respectively, as reported in previous studies on other hard carbons and graphitic‐type carbon samples.^[^ ^10^, ^11^, ^12^, ^19^, ^23^, ^24^, ^25^ ^]^ At higher binding energies (BEs), oxidized carbon species with C─O, C═O, and O─C═O functionalities are observed, with peaks at 286.9, 288.1, and 288.7 eV, respectively. The peak corresponding to species with C–O bonding is the most dominant of these three peaks, as reported in previous literature on hard carbons.^[^ ^11^, ^19^ ^]^ These oxidized carbon species are likely present as a result of the reaction between atmospheric oxygen and moisture with the reactive defects on the surface of the hard carbon particles during electrode preparation. Furthermore, the CMC binder, which is present in the bulk of the hard carbon electrode, contains C–O–C, C–OH, and R–C(═O)–O–Na functionality, which contributes to the C–O and O–C═O peaks. Finally, a peak at 290.7 eV corresponding to the π–π^*^ shake‐up transition which is characteristic of the presence of sp^2^ C, is observed.^[^ ^25^, ^26^ ^]^ The O 1s spectrum (Figure 2b) confirms the presence of oxidized carbon species on the surface of the pristine electrode, with peaks at 530.3, 531.6, and 533.2 eV corresponding to O–Na, C═O/O–C═O and C–O functionality, respectively, with the C–O peak being the most dominant.^[^ ^19^, ^23^ ^]^ Furthermore, the peak in the Na 1s spectrum (Figure S4) centered at 1071.4 eV is attributed to the sodium carboxylate (R–C(═O)–O–Na) groups present in the CMC binder.

### 2.3 HAXPES Data Analysis on Hard Carbon Electrodes After a 10 h OCV Rest

Following a 10 h OCV resting period, distinct compositional changes on the surface of the hard carbon electrodes extracted from half and full cells were observed with respect to each other and the pristine electrode. The C 1s spectrum at 2350 eV (Figure 2a) shows a shift of the sp^2^ C peak to lower eV in the half‐cell electrode (283.9 eV), which can be attributed to partial sodiation (within the graphitic framework) of the hard carbon due to the presence of the Na CE.^[^ ^9^, ^24^ ^]^ At 2350 and 7050 eV (Figures 2 and S5 respectively), both electrodes show a decrease in the relative area of the sp^2^ C peak when compared to the pristine electrode, indicating that the hard carbon electrode is being buried underneath other species present at the surface, implying that a spontaneous SEI layer forms under these conditions. The decrease in the sp^2^ C peak area is more pronounced in the hall‐cell than in the full‐cell, indicating that spontaneous SEI formation is more extensive in the half‐cell sample.

Concomitantly, both electrodes showed an increased contribution from the sp^3^ C peak at 285.0 eV to the C 1s spectra, although this is again more evident in the half‐cell electrode based on peak area calculations. This suggests that a significant proportion of the species within the forming spontaneous SEI contain C–C bonding, i.e., alkyl chains (CH_x_^−^), which are attributed to species formed via EC and DEC electrolyte solvent reduction pathways.^[^ ^27^, ^28^ ^]^ These species include (sodium) alkyl carbonates (e.g., R–OCO_2_Na and R–(OCO_2_Na)_2_), sodium carboxylates (e.g., R–COONa), (sodium) alkoxides (R–ONa) (where R = alkyl groups of varying chain lengths, depending on the decomposition pathway of EC/DEC), and polyolefins ((CH_2_)_n_). These species are thought to be present in the SEI of Li and Na systems when carbonate electrolytes are used.^[^ ^9^, ^28^, ^29^, ^30^, ^31^, ^32^ ^]^

Furthermore, there are significant changes in the amount and type of oxidized carbon species (C═O, O─C═O, and CO_3_^2−^). The C–O and O–C═O peaks observed in the pristine electrode are present in both half‐cell and full‐cell samples. A new peak at 290.2 eV assigned to CO_3_^2−^ species from carbonates such as (sodium) alkyl carbonates and Na_2_CO_3_ is present in both the half‐cell and full‐cell electrodes. Furthermore, the C═O peak observed in the pristine electrode does not appear in the half‐cell OCV sample spectrum. C═O species are unlikely to be found in the SEI since ketones/aldehydes are not considered common SEI components since the surface C═O species tend to react with the electrolyte to form O–C═O and CO_3_^2−^ species.^[^ ^6^, ^9^, ^32^, ^33^, ^34^ ^]^ Subsequently, the absence of the C═O peak further evidences the formation of a spontaneous SEI layer that covers the hard carbon electrode, which does not occur to the same extent in the full‐cell.

The total content of oxidized species in both types of electrodes differs from that of the pristine material, with the half‐cell electrode showing a greater peak area intensity than the full‐cell electrode (Figure 2d). At 7050 eV, an analogous behavior is observed, albeit to a lesser extent (Figure S5d), indicating that these changes occur primarily on the hard carbon electrode surface. The increase of oxidized carbon species likely represents the formation of organic SEI components arising from electrolyte decomposition such as (sodium) alkyl carbonates and carboxylates (e.g., R–O–C(═O)–O–Na, R–(O–C(═O)–O–Na)_2_ and R–C(═O)–O–Na), sodium alkoxides (R–CH_2_–O–Na) and oligomers of oxygen‐containing polymeric species such as polyethylene oxide (–CH_2_–CH_2_–O)_n_.^[^ ^9^, ^27^, ^32^, ^35^, ^36^, ^37^, ^38^ ^]^ The most commonly reported of the aforementioned species is sodium ethylene dicarbonate (NEDC), which results from EC reduction.^[^ ^9^, ^32^, ^39^ ^]^ NEDC is highly soluble in the electrolyte and is thought to react with traces of water to produce Na_2_CO_3_, similarly to lithium ethylene dicarbonate (LEDC) in LIBS.^[^ ^37^, ^40^, ^41^ ^]^ The O 1*s* spectra (Figure 2b) corroborate the data from the C 1s spectra, showing a clear resemblance between the full‐cell and pristine electrode.

The F 1s spectra (Figure 2c) show a peak at 687.8 eV that corresponds to the decomposition products of the NaPF_6_ salt, i.e., Na_x_PF_y_ and Na_x_PF_y_O_z_,^[^ ^9^, ^10^, ^32^ ^]^ in both electrodes. However, it is worth noting that any residual NaPF_6_ salt on the electrode surface may appear at similar BEs and thus contribute to this peak. Due to the similar BEs of the NaPF_6_ decomposition products, this peak cannot be reliably differentiated into specific components. The peak is much broader and has a lower absolute intensity in the full‐cell electrode, as evidenced by the lower signal‐to‐noise ratio. This is particularly evident in the spectra collected at 7050 eV (Figure S5c). A similar observation can be made in the Na 1s and P 2p spectra (Figures S4 and S8), with a lower signal‐to‐noise ratio, observed for the full‐cell OCV sample, indicating a lower concentration of sodium and phosphorus species. Furthermore, the P 2p spectra of the half‐cell OCV sample show two peaks at ≈ 137.7 and ≈ 133.3 eV assigned to P–F bonding (present in Na_x_PF_y_ species) and P–O bonding (present in Na_x_PF_y_O_z_ species), respectively, while the full‐cell OCV sample only shows the former peak. The Na 1s spectrum of the full‐cell OCV sample has a tail to high BE that is not present in the half‐cell, indicating a wider distribution of Na_x_PF_y_ species than the half‐cell electrode. Although difficult to quantify, the NaPF_6_, Na_x_PF_y_, and Na_x_PF_y_O_z_ species are present in varying amounts (except for the latter species, which are most likely not present in the full‐cell), and the absolute amount of these species is significantly lower in the full‐cell. Furthermore, a peak at 684.4 eV in the F 1s spectra, corresponding to NaF formed from the reductive decomposition of NaPF_6_^[^ ^9^, ^10^, ^32^ ^]^ was only present in the half‐cell electrode, further evidencing a more accelerated decomposition of the NaPF_6_ salt in the half‐cell during the 10 h OCV period.

Figure 2e shows the calculated atomic concentration (in at. %) for the pristine and OCV samples at 2350 eV. The data show that the half‐cell electrode has significantly different elemental concentrations on the surface of the hard carbon electrode, whereas the full‐cell electrode looks more similar to the pristine electrode. In both cases, the decrease in sp^2^ C concentration is primarily due to increases in the Na, F, P, and O atomic concentrations, which are accompanied by an increase in the concentration of carbon species other than sp^2^ C. Analogous changes were observed at 7050 eV (Figure S5e), albeit to a lesser extent, due to the greater contribution to the spectrum of the bulk hard carbon electrode, thus further emphasizing that these changes are occurring primarily at the hard carbon electrode surface.

### 2.4 HAXPES Data Analysis on Hard Carbon Electrodes after 1^st^, 2^nd^, and 10^th^ Sodiation Cycles

**Figure 3:**
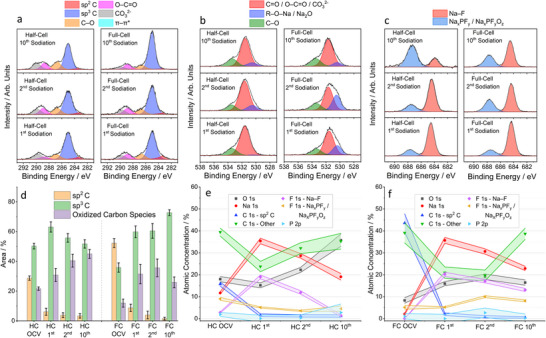
Ex situ HAXPES data obtained at 2350 eV. a) C 1s, b) O 1s, and c) F 1s spectra for hard carbon electrodes extracted from half‐cells (HC) and full‐cells (FC) after the 1^st^, 2^nd^, and 10^th^ cycles (discharge state). d) Area percentage contributions of the sp^2^ C, sp^3^ C, and oxidized carbon species (C–O, C═O, O–C═O, and CO_3_^2−^) to the C 1s spectra fittings. Error bars indicate ±2σ of calculated values. e and f) Calculated atomic concentrations of the elements/compounds (in at. %) and associated errors observed on the surface of the hard carbon electrodes removed from e) half‐cells and f) full‐cells after a 10 h OCV period and the 1^st^, 2^nd^, and 10^th^ sodiation cycles. The plotted errors represent ± 2*σ*, where *σ* is the standard deviation in the fitted peak areas/atomic concentration calculated from the set of Monte Carlo simulations (refer to Note S1.2 in the Supporting Information).

**Figure** **3**a shows the C 1s spectra of the half and full‐cell electrodes measured at 2350 eV after the 1^st^, 2^nd^, and 10^th^ sodiation cycles. After the 1^st^ discharge, both electrodes show a similar spectrum, indicating that the formed SEI consists of equal carbon species. Even though the total contribution of oxidized carbon species to the C 1s spectra was similar in both cases (Figure 3d), the relative contribution of the CO_3_^2−^ and O─C═O peaks to the spectra differed, with the full‐cell electrode showing a greater relative amount of (sodium) alkyl carbonates vs. Na_2_CO_3_ on the surface of the SEI, whereas the half‐cell electrode showed the opposite trend. This could be attributed to the fact that (sodium) alkyl carbonates, which were found to form spontaneously in the half‐cell electrode, may have had more time to decompose and react with traces of water in the electrolyte to form Na_2_CO_3_, resulting in a higher concentration of Na_2_CO_3_ in the SEI of the half‐cell electrode compared to the full‐cell electrode. When examining both electrodes, no significant differences in SEI thickness are observed since the area contribution of the sp^2^ C peak to the total area of the C 1s spectra is similar.

Figures 3b and S6b show the O1s spectra at 2350 eV and 7050 eV, respectively. At both probed energies, the SEI formed in the full‐cell electrode is dominated by the R–O–Na/Na_2_O peak after the 1^s^ ^t^ sodiation cycle. For both the half‐cell and the full‐cell electrode samples, the R–O–Na/Na_2_O peak shows a greater contribution at 7050 eV than at 2350 eV. The R–O–Na/Na_2_O peak can be attributed to sodium alkoxides (R–O–Na) and sodium oxide (Na_2_O). By analogy to Li_2_O in Li‐based systems, Na_2_O has been reported to be present in the NIB SEI.^[^ ^23^, ^42^, ^43^, ^44^ ^]^ In contrast to Na_2_O, sodium alkoxides, if present, would contribute to the C–O peak in the C 1s spectrum. The electrode in the full‐cell has a lower contribution from the C–O peak in the C 1s spectra compared to the half‐cell, indicating that its SEI contains less sodium alkoxides. As a result, the increased contribution of the R–O–Na/Na_2_O peak in the O 1s spectra of the full‐cell electrode compared to the half‐cell electrode can be attributed to Na_2_O rather than R–O–Na groups found in sodium alkoxides. Furthermore, the increase in relative intensity of the R–O–Na/Na_2_O peak upon increasing X‐ray energy suggests that the Na_2_O is present deeper into the SEI and/or that the C–O–Na groups of the CMC binder are contributing more, as evidenced in the O 1s spectrum of the pristine electrode. This trend persists after the 2^nd^ sodiation process; however, after the 10^th^ sodiation cycle, the relative contribution of the O–Na/Na_2_O peak to the spectra becomes comparable in both half‐cell and full‐cell samples. This convergence may be attributed to a significant increase in oxidized carbon species—including sodium alkoxides—in the SEI of the half‐cell electrode between the 2^nd^ and 10^th^ sodiation cycles. This change is less pronounced in the full‐cell.

After the 1^st^ sodiation cycle, the F 1s spectra at 2350 eV (Figure 3c) of both electrodes show Na_x_PF_y_/Na_x_PF_y_O_z_ and NaF peaks with similar relative intensities, indicating that the half‐cell and the full‐cell electrodes contain comparable fluorine‐based species. Significant changes have occurred in comparison to the F 1s spectra of the OCV electrodes (Figure 2c), with the NaF peak being more intense than the Na_x_PF_y_/Na_x_PF_y_O_z_ peak for both electrodes, indicating substantial NaPF_6_ decomposition during the first discharge. The Na 1s spectra (Figure S4a) of the half‐cell and full‐cell samples are also similar, with a single broad peak shifted to lower energies compared to the OCV samples. This shift toward lower energies indicates the presence of more sodium carbonate and/or organic sodium‐containing species, such as sodium alkoxides and (sodium) alkyl carbonates. Figures 3e,f and S7 show the atomic concentration of elements at 2350 and 7050 eV in the cycled half‐cell and full‐cell samples. Corroborating the above findings, the half‐cell and full‐cell samples after the 1^st^ sodiation cycle show similar concentrations of the elements present in their respective SEIs, with no significant differences observed.

After 10 cycles, differences in the evolution of the SEI chemical composition emerge between the two electrodes. As cycling progresses, the SEI formed in the half‐cell electrode contains more sodium carbonate and organic species such as (sodium) alkyl carbonates, carboxylates, and sodium alkoxides. This is demonstrated by an increase in the relative contribution of the C─O, O─C═O, and CO_3_^2−^ peaks in the C 1s spectra at both energies (Figure 3a and Figure S6a), as well as a consistent increase in the atomic concentration of oxygen from the 1^st^ to the 10^th^ sodiation cycle at 2350 and 7050 eV (Figure 3e and Figure S7, respectively). In contrast, in the full‐cell electrode, the atomic concentration of oxygen and the relative amount of oxidized carbon species in the C 1s spectra remain similar from the 1^st^ to the 10^th^ cycle at both energies (Figure 3d,f; Figures S6d and S7b). However, for the full‐cell electrode, cycling increases the relative contribution of the sp^3^ C peak (attributed to hydrocarbons/polyolefins within the SEI).^[^ ^29^ ^]^

Both electrodes show SEI thickening during cycling, as evidenced by a decrease in the relative contribution of the sp^2^ C peak to the C 1s spectra measured at 7050 eV (Figure S6a,d), although the SEI is consistently thinner (larger contribution from the sp^2^ C peak) in the full‐cell electrode at all cycling stages. At 7050 eV, the sp^2^ C peak contributes 22.8 ± 1.4 %, 18.9 ± 0.9 %, and 12.0 ± 0.8 % for the half‐cell samples and 25.9 ± 0.8 %, 22.2 ± 1.2 %, and 15.5 ± 1.5 % for the full‐cell samples at the 1^st^, 2^nd^, and 10^th^ sodiation cycles, respectively.

The O 1s spectra of the full‐cell electrodes show a significant decrease in the relative contribution of the R–O–Na/Na_2_O peak from 29.8 ± 2.7 % to 8.6 ± 1.3 % from the 2^nd^ to the 10^th^ cycle compared to the half‐cell electrode. This decrease is not accompanied by a significant decrease in the relative contribution of the C–O peaks in the C 1s spectra, indicating that Na_2_O is primarily responsible for the R–O–Na/Na_2_O peak in the O 1s spectra of the full‐cell samples. The observed decrease in the R–O–Na/Na_2_O peak indicates that the Na_2_O species in the SEI of the full‐cell electrode are being covered by other SEI species to a greater extent than in the half‐cell electrode during further cycling, likely due to the increase in polyolefins.

The F 1s spectra (Figure 3c and Figure S6c) show that the Na–F peak decreases with respect to the Na_x_PF_y_/Na_x_PF_y_O_z_ peak for the half‐cell electrode samples but remains similar for the full‐cell electrode samples, with only a minor decrease observed during cycling. Furthermore, the atomic concentration of Na_x_PF_y_/Na_x_PF_y_O_z_ species remains relatively constant with cycling in both electrodes, with no clear trend, whereas the atomic concentration of NaF decreases in both the half‐cell and full‐cell samples, with the half‐cell showing a greater decrease. It is worth noting that the changes in the atomic concentration of sodium species during cycling follow a similar pattern to those observed for NaF concentration.

The orange and purple regions in the Na 1s spectra (Figures S4a and S8a) indicate the presence of Na_2_CO_3_ and organic (R–O–Na/–C(═O)O–Na) and inorganic (Na_x_PF_y_/Na_x_PF_Y_O_z_/NaF) sodium‐containing species. After 10 cycles, the peak in the Na 1s spectra shifts to a lower BE relative to the 1^st^ sodiation cycle for the half‐cell electrode, indicating the presence of organic Na‐containing species. In contrast, the full‐cell electrode shows a shift to higher BE, indicating the presence of inorganic Na‐containing species. This contrasting peak shift supports previous observations of a more organic‐rich SEI evolving with cycling in the half‐cell electrode compared to the full‐cell electrode.

In summary, during the OCV resting period, changes in the C 1s and O 1s spectra relative to the pristine electrode indicate that an SEI layer is forming spontaneously on the surface of the hard carbon electrode in the half‐cell, with minimal changes observed in the full‐cell. The surface layer formed at OCV in the full‐cell electrode is significantly thinner than in the half‐cell electrode and is primarily made up of hydrocarbons found in polyethylene oxide oligomers, polyolefins, and sodium alkoxides. In contrast, the SEI that forms in the half‐cell electrode contains both organic and inorganic species. The organic species are primarily carbon and oxygen‐containing species such as (sodium) alkyl carbonates and carboxylates, which are most likely present as a result of the decomposition of the EC and DEC electrolyte solvents through a similar mechanism to that reported in Li‐ion systems.^[^ ^9^, ^27^, ^32^, ^35^, ^36^, ^37^, ^38^ ^]^ Inorganic species present include NaPF_6_ decomposition products such as Na_x_PF_y_, Na_x_PF_y_O_Z_, and NaF.^[^ ^45^ ^]^ Na_2_CO_3_ is also present in the half‐cell OCV sample (as evidenced by the CO_3_^2−^ peak in the C 1s spectra). Na_2_CO_3_ is a byproduct of EC and/or DEC decomposition, but it is also thought to form as a result of the reaction of (sodium) alkyl carbonates with any H_2_O impurities present in the electrolyte, similar to that reported for Li_2_CO_3_ in Li‐ion systems.^[^ ^46^ ^]^

Na metal CE is responsible for SEI formation in the half‐cell OCV sample. The Na metal CE reacts spontaneously with the carbonate‐based electrolyte, forming its own SEI, as shown in previous studies.^[^ ^2^, ^4^, ^7^ ^]^ The species formed on the surface of the Na metal are likely to diffuse throughout the cell and deposit on the surface of the hard carbon working electrode, which explains the formed SEI at OCV in the half‐cell electrode. This behavior is consistent with previous studies using Na_2_Ti_3_O_7_ and Cu_2_Sb electrodes in sodium half‐cells.^[^ ^6^, ^47^ ^]^ The surface chemistry of the hard carbon electrode in the half‐cell, as well as the chemistry of the electrolyte, were significantly altered during the 10 h OCV rest period as a result of spontaneous decomposition at the surface of the Na metal CE. As a result, SEI formation and its subsequent evolution during galvanostatic cycling in the half‐cell is likely to differ from that observed in the full‐cell, where spontaneous SEI formation was not as prominent.

Following the first discharge process, the chemical composition of the SEI formed in the half‐cell and the full‐cell electrodes is similar. However, as cycling progresses, the chemical compositions of the SEIs become more distinct. The half‐cell samples show a steady increase in the content of oxygen and oxidized carbon species, which coincides with a decrease in the relative content of NaF in the SEI. This decrease in NaF content is most likely due to an increase in the deposition of carbon and oxygen‐containing organic species within the SEI of the half‐cell electrode as cycling progressed, resulting in a faster rate of SEI growth for the half‐cell and, as a result, burying the NaF at a faster rate than in the full‐cell. In contrast to the half‐cell samples, the overall content of oxidized carbon and oxygen‐containing species within the SEI of the full‐cell samples is more stable, with the SEI being richer in inorganic species and hydrocarbons/polyolefins as cycling progresses relative to the half‐cell. Furthermore, during cycling, the SEI in the full‐cell electrode is thinner than in the half‐cell electrode.

### 2.5 ToF‐SIMS

**Figure 4:**
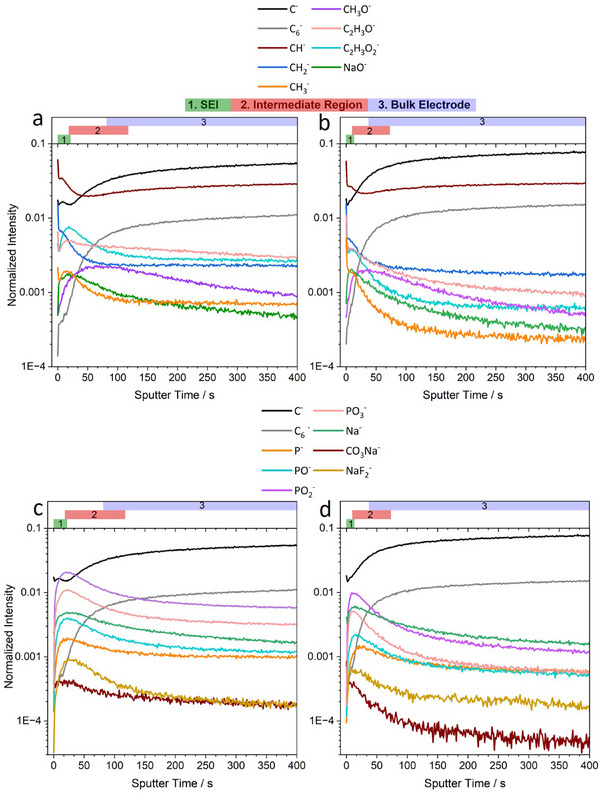
ToF‐SIMS negative ion depth profiles of selected organic (top) and inorganic (bottom) secondary ion fragments after the 10^th^ sodiation cycle for a, c) half‐cell electrodes and b, d) full‐cell electrodes.

ToF‐SIMS in negative‐ion mode was performed on the hard carbon pristine electrode (Figure S9) and the electrodes were extracted from the half‐cell and full‐cell after 10 cycles in the discharged state. Depth profiling was performed by acquiring sequential mass spectra after a sputtering period. The figures show the normalized intensity of selected secondary ion fragments for organic species (**Figure** **4**a,b) and inorganic species (Figure 4c,d). The intensity of the fragments is plotted against sputtering time, representing increasing depth into the electrode. These specific fragments were chosen to represent the species hypothesized to be present within the SEI based on HAXPES spectra analysis and previous ToF‐SIMS work on the SEI formed in Li and Na systems using carbonate‐based electrolytes similar to those used in this study.^[^ ^48^, ^49^, ^50^, ^51^, ^52^, ^53^, ^54^, ^55^ ^]^

The C^−^ and C_6_^−^ fragments were chosen to represent the hard carbon electrode because they appear prominently in the depth profile of the pristine electrode (Figure S9). These have previously served as markers for graphite electrodes.^[^ ^50^, ^56^, ^57^, ^58^ ^]^ Carbonyl‐containing species such as (sodium) alkyl carbonates and carboxylates are represented by the C_2_H_3_O^−^, C_2_H_3_O_2_^−^, and CH_3_O^−^ fragments, while alkyl chains found in all organic species are represented by the CH^−^, CH_2_^‐^_,_ and CH_3_^‐^ fragments.^[^ ^48^, ^49^, ^50^ ^]^ The NaO^‐^ fragment represents sodium oxide, sodium alkoxides, (sodium) alkyl carbonates, and carboxylates with O‐Na bonds.^[^ ^49^, ^50^, ^54^ ^]^ The P^−^, PO^−^, PO_2_^−^, PO_3_^−^, Na^−^, CO_3_Na^−^ and NaF_2_^−^ fragments have been selected to represent inorganic compounds hypothesized to be present, such as Na_x_PF_y_O_z_, Na_2_CO_3_, and NaF.^[^ ^49^, ^50^, ^55^ ^]^

The concentrations of C^−^ and C_6_^−^ fragments increase quickly in the pristine electrode before stabilizing, as sputtering continues. Thus, the C^−^ and C_6_^‐^ fragments make up most of the hard carbon electrode. This contrasts with all the other fragments presented for the pristine sample, which initially decreases in concentration at the start of the sputtering process before stabilizing. This means that all fragments except C^‐^ and C_6_^‐^ have a higher concentration at the surface of the hard carbon electrode than in the bulk. Thus, it is likely that these fragments are caused by defects on the surface of hard carbon particles, such as oxygen functionality (as demonstrated in the HAXPES data of the pristine electrode, i.e., C─O, C═O, and O─C═O peaks in the C 1*s* and O 1s spectra), as well as dangling carbon bonds from graphene layer edges, which result in C_x_H^‐^ fragments.^[^ ^57^ ^]^ Since the C^‐^ and C_6_^‐^ fragments should be primarily present in the pristine electrode, they can be used to determine the thickness of the formed SEI. Therefore, Figure 4 shows the C^‐^ and C_6_^‐^ fragments in the ToF‐SIMS depth profile plots of the half‐cell and full‐cell samples. 

In Figure 4, Region 1 is designated as the SEI, and it contains the maximum signal intensity of most of the fragments. Region 2 is designated as the intermediate region; it represents the region over which the intensity of fragments representing SEI species typically begins to rapidly decrease. Region 3 is designated as the bulk electrode; this is the region where the SEI has been etched away and only the bulk electrode is being probed. The region boundaries are difficult to determine accurately from the fragments shown in Figure 4; thus, some overlap between regions exists. After 10 cycles, the intensity profiles of the C^‐^ fragment in the half and full‐cell electrodes show a different trend than that of the pristine electrode. The signal intensity of the C^‐^ fragment remains constant within Region 1 of the figure. This behavior can be attributed to the SEI present on the surface of both the half‐cell and full‐cell cycled samples, which have a lower concentration of C^‐^ than the bulk hard carbon electrode. As sputtering progresses and the SEI is gradually etched away, the C^‐^ fragment present primarily in the bulk electrode begins to contribute more significantly to the signal, as indicated by the increase in the signal intensity of the C^‐^ fragment in Region 2 marked in the figure, which then begins to stabilize as it moves to Region 3. A similar trend can be observed for the C_6_^‐^ fragment.

Within Region 1, the half‐cell and full‐cell samples show the same general trends in the evolution of fragment intensity with respect to sputtering time, with the fragments showing either one of two trends: 1) A rapid decrease in signal intensity over the first 3–4 s of sputtering, which then stabilizes or increases again to form a secondary maximum in intensity, e.g., fragments CH^‐^, CH_2_^‐^, CH_3_^‐^, C_2_H_3_O^‐^, and C_2_H_3_O_2_^‐^; and 2) an increasing signal intensity during Region 1, e.g., organic fragments NaO^‐^ and CH_3_O^‐^, and all inorganic fragments. The trends observed in Region 1 can be explained by an SEI with two strata. At the surface level, the SEI in both the half‐cell and full‐cell electrodes contains a high concentration of non‐sodium organic alkyl carbonates with long alkyl chains. This observation is supported by the high initial signal intensity of the CH^‐^, CH_2_^‐^, CH_3_^‐^, C_2_H_3_O^‐^, and C_2_H_3_O_2_^‐^ fragments, whereas the content of P‐ and F‐containing inorganic species and Na‐containing organic species, such as alkoxides, is low. This phenomenon is evidenced by the low initial signal intensities of the NaO^‐^ and CH_3_O^‐^ organic fragments and all the inorganic fragments. However, as sputtering begins and the SEI is etched away, a second stratum emerges, rich in all types of organic and inorganic species, with maxima visible in all plotted fragments. This distinct structure, visible for both the SEI in the half‐cell and full‐cell samples, was not apparent in the HAXPES spectra. This discrepancy is most likely due to the fact that in the HAXPES spectra, even at 2.35 keV, the entire depth of the SEI is probed, as the sp^2^ C peak, while small, remains visible in the C 1s spectra after the 10^th^ sodiation cycle of both samples. In contrast, the ToF‐SIMS data probes only 1–2 nm of the surface between sputtering periods. This interpretation of the SEI is consistent with the widely accepted mosaic SEI model introduced by Peled et al.,^[^ ^59^, ^60^ ^]^ in which the SEI is composed of a mosaic of polyhetero microphases of both organic and inorganic components, with the inner SEI having a higher concentration of inorganic components and the outer SEI being more porous and containing a higher concentration of organic species.^[^ ^29^ ^]^

Despite the similarities, Regions 1 and 2, representing the SEI and the intermediate region, respectively, are more extensive in the half‐cell sample (end of Region 1 ≈ 21.9 s and end of Region 2 ≈ 117.7 s) than in the full‐cell sample (end of Region 1 ≈ 13.3 s and end of Region 2 ≈ 73.2 s). This is evidenced by the half‐cell fragments showing a smaller decrease in signal intensity with respect to sputter time in region 2 than analogous fragments in the full‐cell sample. This suggests that the SEI formed in the half‐cell is thicker than the SEI formed in the full‐cell, which supports the HAXPES C 1s data at 7050 eV. Furthermore, the signal intensity of the CH^‐^, CH_2_^‐^, C_2_H_3_O^‐^, and C_2_H_3_O_2_^‐^ fragments near the end of Region 1 are more intense in the half‐cell sample than in the full‐cell sample. This observation supports the HAXPES data after the 10^th^ sodiation cycle, indicating that the half‐cell electrode has a higher SEI content in organic carbonates than the full‐cell.

**Figure 5:**
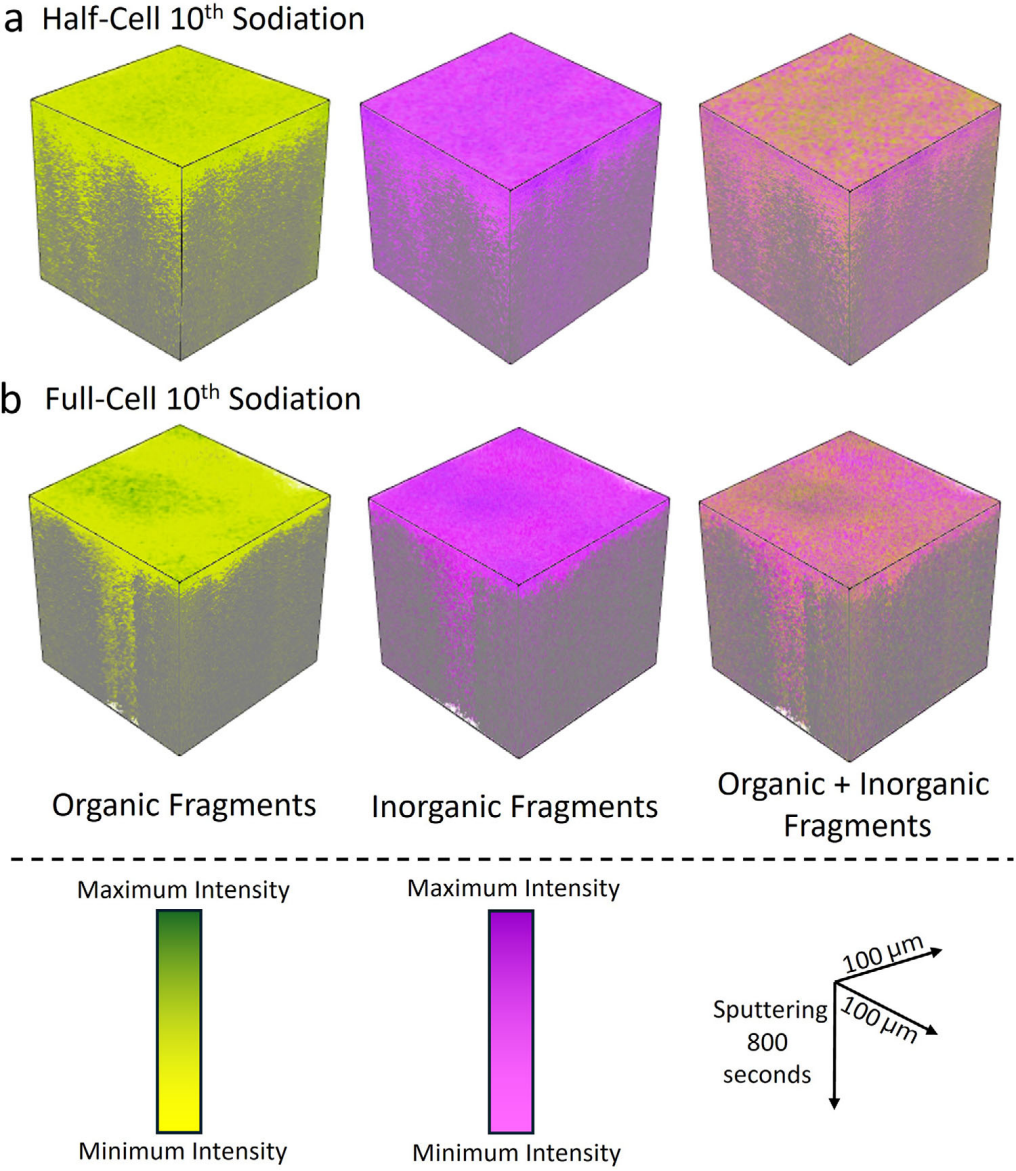
3D image reconstruction from the ToF‐SIMS depth profiling experiments over 100 µm (x‐axis) by 100 µm (y‐axis) raster size and 800 s of total sputtering time (z‐axis) of selected secondary ion‐fragments after the 10^th^ sodiation cycle for a) half‐cell and b) full‐cell electrodes. The left‐hand column shows selected organic fragments: C_2_H_3_O^‐^, C_2_H_3_O_2_^‐^, CH_3_O^−^, and NaO^−^. The middle column shows selected inorganic fragments: P^−^, PO_2_^−^, PO_3_^−^, NaF_2_^−^, CO_3_Na^−^, and Na^−^. The right‐hand column shows both the organic and inorganic fragments combined. The C^−^ fragment, which represents the hard carbon electrode, is shown in grey in all the images.

This is also evident in **Figure** **5**, which shows 3D images reconstructed from ToF‐SIMS depth profiles of the half‐cell and full‐cell 10^th^ sodiation cycle samples of selected organic (C_2_H_3_O^‐^, C_2_H_3_O_2_^‐^, CH_3_O^‐^, and NaO^‐^) and inorganic (P^‐^, PO_2_^‐^, PO_3_^‐^, NaF_2_^‐^, CO_3_Na^‐^, and Na^‐^) fragments, as well as a combination of both. The half‐cell sample has a noticeably thicker SEI in both organic and inorganic species than the full‐cell. Furthermore, when the organic and inorganic fragments are combined into a single image (right‐hand column of Figure 5), a higher concentration of organic species is observed on the surface of the SEI in the half‐cell sample, supporting the formation of a more organic‐rich outer layer in the half‐cell than in the full‐cell. Figure 5 shows that SEI species (both organic and inorganic species) are found at greater depths within the electrode in certain areas. This is most likely attributed to SEI formation in pores/cracks present in the electrode, as SEI species are typically found where the C^‐^ fragment is at its lowest. The half‐cell sample has higher maximum signal intensities of P^‐^, PO^‐^, PO_2_^‐^, PO_3_^‐^, and NaF_2_^‐^ fragments than the full‐cell sample, as shown in Figure 4c,d. This observation contradicts the findings of the HAXPES data, which show that the full‐cell electrode after the 10^th^ sodiation cycle contained more inorganic species than the half‐cell electrode. This discrepancy may be attributed to the use of sputtering in the ToF‐SIMS experiment. Previous studies have shown that sputtering of the SEI can change its composition, with Li_x_PF_y_ species decomposing to form LiF and Li_x_PF_y_O_z_ species as sputtering progresses.^[^ ^61^ ^]^ As a result, any differences in the amount of NaP_x_F_y_ species on the electrode surface would cause variations in the amount of NaF and Na_x_PF_y_O_z_ species formed during sputtering.^[^ ^67^ ^]^ Additionally, sputtering is known to preferentially remove organic SEI components over inorganic compounds.^[^ ^62^ ^]^ Therefore, while the HAXPES analysis revealed that the SEI in the full‐cell electrode after the 10^th^ sodiation cycle is relatively richer in inorganic species, the SEI in the half‐cell is thicker overall, resulting in a higher absolute amount of all SEI species in the half‐cell, as shown in Figure 5. This implies that if the organic SEI components are preferentially sputtered away, there will be a significantly higher relative content of inorganic species observed at any given time.

## 3 Conclusion

HAXPES data analysis revealed similar chemical compositions of the SEIs formed in half and full‐cells after the 1^st^ sodiation (discharge) process. Both exhibited oxygen‐containing organic functionalities, i.e., (sodium) alkyl carbonates and carboxylates, and fluorine‐containing inorganic functionalities (i.e., NaF and Na_x_PF_y_O_z_), which are typical of carbonate solvents and NaPF_6_ salt decomposition, respectively. However, as cycling progressed, significant differences in the evolution of the chemical composition of the SEI became apparent. Specifically, the SEI formed in the half‐cell showed a steady increase in the relative concentration of organic species within it. This coincided with a decrease in the relative content of inorganic species, resulting in a thicker SEI. This contrasts with the SEI formed in the full‐cell, which had a more stable chemical composition during cycling, with only minor changes in the relative content of oxygen‐containing organic and fluorine‐containing inorganic species as cycling progressed. Overall, this resulted in an SEI with a significantly higher relative inorganic content than that observed in the half‐cell after several cycles. ToF‐SIMS measurements confirmed these findings, revealing a thicker SEI with a higher proportion of carbon‐ and oxygen‐containing organic fragments in the half‐cell, characteristic of sodium/organic alkyl carbonates and carboxylates, and sodium alkoxide species (e.g., CH^−^, CH_2_^−^, CH_3_^−^, C_2_H_3_O^−^, C_2_H_3_O_2_^−^, CH_3_O^−^, and NaO^−^).

These results suggest that in half‐cells with hard carbon as the negative electrode, the presence of a Na metal CE triggers the formation of an SEI, which becomes increasingly rich in oxygen and carbon‐containing organic species as cycling progresses. This is most likely due to the formation of (sodium) alkyl carbonates and carboxylates, which are believed to be a primary component of the NIB SEI formed in carbonate‐based electrolytes.^[^ ^9^, ^38^, ^39^ ^]^ The presence of a Na metal CE was found to cause the spontaneous formation of an SEI at the hard carbon. This spontaneous SEI is made up of organic species from EC/DEC reduction (e.g., (sodium) alkyl carbonates and carboxylates, sodium carbonate, and polymeric oxygen species like polyethylene oxide), as well as inorganic species from NaPF_6_ salt reduction (e.g., NaF and Na_x_PF_y_O_z_). This spontaneous SEI on the hard carbon electrode is caused by the leaching of reductive decomposition species present on the surface of the Na metal, which is known to react spontaneously with carbonate electrolytes.^[^ ^2^, ^4^, ^5^, ^6^ ^]^ Thus it is possible that when current is applied during galvanostatic cycling, the reactions between the Na metal and the electrolyte are catalyzed and begin to occur at a significantly faster rate, influencing SEI formation at the hard carbon electrode. It is well established that the interphase formed on the surface of Na metal after electrochemical cycling is unstable, resulting in a continuous increase in cell polarisation and eventually, failure of Na:Na symmetrical cells.^[^ ^2^ ^]^ Furthermore, organic species within the SEI, such as (sodium) alkyl carbonates (e.g., NEDC) and carboxylates, and sodium alkoxides, are more susceptible to dissolution than inorganic species such as NaF and Na_2_CO_3_, due to their similar polarity to the carbonate solvents.^[^ ^63^ ^]^ This occurrence has been observed to be particularly pronounced in Na‐based systems compared to analogous Li systems.^[^ ^3^, ^64^, ^65^, ^66^ ^]^ Consequently, if species such as sodium carboxylates form on the Na metal surface, a significant proportion is likely to dissolve back into the electrolyte, permeate throughout the cell, and continuously deposit onto the hard carbon electrode surface. This mechanism could explain the substantial and persistent increase in oxidized carbon species in the SEI formed in the half‐cell during cycling compared to the full‐cell.

It is worth noting that in the full‐cell, a cathode electrolyte interphase (CEI) will have formed due to electrolyte oxidation at the surface of the cathode material, caused by the high voltage experienced by the cathode material during cycling (≈ 4.3 V). As a result, the CEI may influence the composition and thickness of the SEI at the hard carbon anode. Crosstalk occurring between the cathode and anode is supported by the widespread observation of transition metal (TM) dissolution from cathode materials into the electrolyte, which is then deposited onto the SEI layer forming at the anode.^[^ ^67^, ^68^, ^69^, ^70^ ^]^ TM dissolution is particularly frequent for Mn ions, resulting in capacity loss over long‐term cycling in full‐cells.^[^ ^71^ ^]^ There was no evidence of TM dissolution happening across the 10 cycles evaluated in this study since no TM‐related peaks were observed in any of the survey scans performed on the full‐cell samples (Figures S2 and S3). However, it is plausible that the CEI influences the creation of the SEI, and vice versa. The link between CEI stability and SEI composition is a clear avenue for future research.

These findings highlight the significance of transitioning from sodium half‐cell to full‐cell investigations when developing our understanding of the SEI. Most previous studies on the NIB SEI formed on hard carbon anodes have been conducted exclusively using half‐cells. Therefore, this work challenges our current understanding of the NIB SEI at hard carbon anodes. Recent studies comparing the SEI formed at hard carbon anodes in half‐cells using carbonate‐based or ether‐based electrolytes are one example of how these findings are likely to have an impact on our current understanding of the SEI.^[^ ^43^, ^44^, ^72^, ^73^, ^74^ ^]^ These studies have shown that the SEI formed in the diglyme electrolyte is more uniform, thinner, and richer in inorganic species such as NaF and Na_2_O than that found in the carbonate‐based electrolyte. These findings were used to explain the improved electrochemical performance of the half‐cell when using the diglyme electrolyte over the carbonate‐based electrolyte. However, a recent study found that when carbonate and diglyme‐based electrolytes are used in a full‐cell, their performance is remarkably similar, in contrast to the half‐cell observations.^[^ ^75^ ^]^ This suggests that the differences in SEI observed in half‐cells may be primarily caused by the increased reactivity of carbonate solvents with the Na metal CE. This emphasizes the importance of using full‐cells to determine SEI behavior, especially when using carbonate‐based electrolytes, which, as demonstrated in this work, can have a significant impact on the chemical composition and subsequent properties of the formed SEI at the anode material.

## 4 Experimental Section

### Electrochemical Characterization

Deregallera (United Kingdom) provided biowaste‐derived hard carbon that had been carbonized at 700 °C for this study. Electrodes were made from a slurry consisting of hard carbon as the active material and a mixture of sodium carboxymethyl cellulose (CMC, Ashland) and styrene butadiene rubber (SBR, MTI, 50 wt.% in aqueous solution) as the binder, in a 95:2.5:2.5 wt.% ratio. To prepare the slurry, 0.5 g of the solid mixture were dissolved in 0.75 g of deionized water (MilliQ) and mixed at 1800 rpm using a Thinky mixer (ARE‐250, Intertronics) for 30 min to form a viscous slurry. This mixture was cast onto an aluminum foil (16 µm thick, 99.45% purity, TOB New Energy) at a controlled speed of 5 mm s^−1^ using a doctor blade with a 100 µm aperture and an automatic film coating machine (TOB‐VFC‐150, TOB New Energy). The resulting coating was left to dry in a fumehood for 1 h before being transferred to a drying oven at 80 °C and left to dry overnight. A hydraulic punch (TOB‐CP60, TOB New Energy) was used to punch out 12 mm diameter electrode discs from the foil. The electrode discs were dried at 80 °C under vacuum before being transferred to an argon‐filled glovebox (H_2_O and O_2_ < 0.1 ppm, MBraun). The active material loading on as‐prepared electrodes was 3.20 ± 0.59 mg cm^−2^ (based on an average mass of 49 electrodes from the coating used).

Electrochemical testing was performed in a two‐electrode coin cell configuration (Na half‐cell) and a three‐electrode PAT cell (EL‐CELL) (Na full‐cell), as shown in Figure S1. Cell assembly was conducted inside an argon‐filled glovebox. For the two‐electrode coin cell preparation, hard carbon was used as the WE and a 15 mm Na metal disc (ingot, 99.8% metal basis, Alfa Aesar) as the CE and reference electrode (RE).

For the three‐electrode full‐cell preparation, 12 mm diameter hard carbon electrodes were used as the CE/anode, 12 mm diameter electrodes consisting of Na_0.79±0.05_Ni_0.27±0.05_Mn_0.42±0.05_Mg_0.15±0.05_Ti_0.17±0.05_O_2±0.05_ active material, polyvinylidene fluoride (PVDF) binder, and carbon black conductive additive in a 92:3:5 (wt.% ratio) were used as the WE/cathode, and a Na metal ring (Alvatek) as the RE. Na metal was the simplest choice for the three‐electrode PAT cell set‐up (EL‐CELL) used in this work; however, Na metal is an unreliable RE due to the reactivity of Na to carbonate‐based electrolytes.^[^ ^14^, ^15^, ^16^ ^]^ The authors acknowledge that that the use of a Na metal RE might have affected the conclusions of this work. This issue is discussed in more detail in Note S1.1 of the Supporting Information. The cathode:anode mass ratio ranged from 2.031 to 2.048 for the full‐cells, resulting in a reversible capacity ratio of ≈ 1.60, based on the 3^rd^ cycle half‐cell discharge capacities of hard carbon and Na_0.79±0.05_Ni_0.27±0.05_Mn_0.42±0.05_Mg_0.15±0.05_Ti_0.17±0.05_O_2±0.05_ at 25 mA g^−1^. An excess of cathode material was used to ensure that the electrochemical performance was comparable to that of the half‐cells, which have an abundant supply of Na^+^ ions for SEI formation and subsequent reformation during cycling.

All cells used two separators: an 18 mm diameter disc of glass fiber (Whatman, GF/F) and a polytetrafluoroethylene (PTFE) film (Celgard). To avoid contamination from the glass fiber separator, the hard carbon electrode was placed in the cells facing the PTFE film. To remove traces of moisture, the glass fiber and PTFE separators were dried overnight at 120 and 60 °C, respectively, before cell preparation. The electrolyte consisted of 1 m sodium hexafluorophosphate (NaPF_6_, 99+%, Fischer Scientific) dissolved in ethylene carbonate (EC, anhydrous, battery grade, Gotion) and diethyl carbonate (DEC, anhydrous, battery grade, Gotion) (1:1 by vol.%). The electrolyte was prepared by adding the NaPF_6_ salt (previously dried under vacuum at 80 °C overnight) into a previously prepared EC and DEC solvent mixture (1:1 by vol.%) in a glass vial and stirring the mixture with a stirring plate at 600 rpm for 24 h. The EC:DEC solvent mixture was dried with molecular sieves (4 Å pore size, Sigma Aldrich) for at least a week before use. The electrolyte solution was left to rest for a day to allow any undissolved particles to settle to the bottom of the vial. These undissolved particles have been observed in the literature during electrolyte preparation when using commercially available NaPF_6_ salt of 99+% purity and identified as a NaF impurity phase.^[^ ^79^, ^80^ ^]^ The concentration of NaPF_6_ in the prepared electrolyte is ≈ 0.8 m. However, it is important to highlight to the reader that the presence of this impurity in the NaPF_6_ salt used could have unknowingly affected the electrochemical performance observed and, as such, the chemical composition and resultant properties of the SEI. The clear solution was then decanted into an aluminum bottle and was allowed to dry in molecular sieves for a week. The electrolyte was then decanted into a new aluminum bottle without molecular sieves for longer‐term storage. This was done to prevent the molecular sieves from absorbing ionic species from the electrolyte for an extended time. During the week of storage in molecular sieves, some ion adsorption might have occurred, lowering the concentration of NaPF_6_ below ≈ 0.8 m. However, since all samples in this study were tested with the same electrolyte batch, the comparative assessment of the chemical compositional differences of the SEIs in the half‐cell vs. full‐cells in this work was unaffected. Before undergoing electrochemical testing, the cells were kept at open circuit voltage (OCV) for 10 h to allow the electrolyte to permeate the separators and hard carbon electrode. The half‐cells were then galvanostatically cycled in the 2.0–0.01 V voltage window at a current rate of 15 mA g^−1^ using a battery cycler (BTS4000, Neware), while the three‐electrode full‐cells were galvanostatically cycled in the 2–0.01 V voltage window at a current rate of 15 mA g^−1^ with respect to the hard carbon anode vs. the Na metal RE, using a potentiostat (VMP‐300, Biologic). The full‐cells were cycled using this electrochemical protocol to ensure that the hard carbon electrode reached a voltage comparable to that of the half‐cell. For more information on the cell setups and electrochemical cycling protocols used in this work, refer to Note S1.1 in the Supporting Information.Data were plotted using OriginLab software package.^[^ ^88^ ^]^

### Hard X‐ray Photoelectron Spectroscopy (HAXPES)

Prior to HAXPES data collection, cells were disassembled in an argon glovebox following i) a 10 h rest time during the OCV stage or ii) the 1^st^, 2^nd^, and 10^th^ sodiation cycles. The hard carbon electrodes were extracted from the cell and washed with 1 mL of DEC to remove any dried electrolyte residue. The electrodes were then left to dry for 15 min on lint‐free tissue before being vacuum dried overnight in the glovebox antechamber. After drying, the electrodes were mounted onto copper sample plates using double‐sided copper tape. The mounted samples were then placed in a transfer suitcase to avoid exposure to air before being transported to the Surface and Interface Structural Analysis beamline (I09) at Diamond Light Source (UK) for measurement.^[^ ^81^ ^]^

Hard X‐ray photoelectron spectroscopy (HAXPES) was performed using excitation energies of 2350 and 7050 eV, monochromatised by a Si(111) double‐crystal. C 1s, O 1s, F 1s, Na 1s, and P 2p spectra were measured for each sample at 2350 and 7050 eV, which correspond to approximate probing depths of ≈ 20 and ≈ 50 nm, respectively. The probing depths correspond to three times the inelastic mean free path (IMFP) of electrons, as determined using the TPP‐2M equation.^[^ ^89^ ^]^ The IMFP values were estimated using parameters for polyethylene (as detailed in the NIST database), which was used as a low‐density material indicative of the surface layer studied here.^[^ ^82^, ^83^ ^]^ These depths should be regarded as upper limits since they imply that electrons flow in a straight line, which is not the case because electrons can change direction due to scattering events. It should also be noted that smaller probing depths are expected for materials denser than polyethylene in the sample, such as the bulk electrode itself and inorganic species in the SEI. The atomic concentrations shown were calculated using relative sensitivity factors derived from the photoionization cross–sections of the relevant subshells calculated by Scofield^[^ ^84^ ^]^ at both 2350 and 7050 eV. The data presented in this work were all measured at 2350 eV, while data measured at 7050 eV are shown in the Supporting Information.

Previous studies have examined the potential of the SEI formed at battery electrodes to undergo radiation beam damage during measurements.^[^ ^85^, ^86^, ^87^ ^]^ To avoid radiation damage to the samples, a representative sample from the series was chosen, and F 1s spectra were collected repeatedly at the same sample location. If there was any change in the F 1s spectrum between measurements (which we attributed to beam damage), the beam was defocused, and the test was performed on a new sample spot. The beam was defocused until no radiation damage was observed after 30 min of exposure, resulting in a final spot size between 300 and 400 µm (H) and up to 1 mm (W). A charge neutralizer was not used during the measurements. A hemispherical VG Scienta EW4000 analyzer was set to pass energies of 70 and 100 eV to record the spectra taken at 2350 and 7050 eV, respectively. Data processing, peak fitting, and Monte Carlo error analysis were performed using the CasaXPS software.^[^ ^90^ ^]^ For further information on the procedure used, peak fitting and Monte Carlo error analysis, refer to Note S1.2 and Tables S1–S9 in the Supporting Information.

### Time‐of‐Flight Secondary Ion Mass Spectrometry (ToF‐SIMS)

For the ToF‐SIMS measurements, cells were disassembled inside an argon‐filled glovebox, and hard carbon electrodes were removed from the cell and washed with 1 mL of DEC to remove the dried electrolyte residue. The electrodes were then placed in vials and left to dry for 15 min on lint‐free tissue followed by vacuum drying in a glovebox antechamber. After drying, the samples were mounted onto a sample plate and placed inside a vacuum transfer suitcase. The suitcase was then transported to the load‐lock chamber of a ToF‐SIMS instrument.

ToF‐SIMS (ToF‐SIMS 5, ION‐ToF GmbH, Münster, Germany) was used to obtain depth profile information on the SEI's chemical composition. A 25 keV Bi^+^ primary ion beam (*I* = 0.31 pA) with an analysis area of 100 × 100 µm^2^ was used in the high current bunch mode (HCBM). Depth profiling of the samples was achieved with a 1 keV Cs^+^ sputter source (*I* = 80 nA) with a sputter area of 300 × 300 µm^2^. All ToF‐SIMS measurements were carried out in negative‐ion mode with an electron flood gun to achieve charge compensation. Data were acquired by repeating the following measuring sequence: one measurement scan, followed by 10 sputter frames, and then a 1 s pause. Data were processed using SurfaceLab (version 7 ION‐ToF, GmbH, Münster, Germany).

## Conflict of Interest

The authors declare no conflict of interest.

## Supporting information



Supporting Information

## Data Availability

The data that support the findings of this study are available from the corresponding author upon reasonable request.
