# Supplementary material for: An in‐depth Study of the Solid Electrolyte Interphase Compositional Evolution in Sodium‐Ion Batteries: Unravelling the Effects of a Na Metal Counter Electrode on the SEI
Source: Adv Sci (Weinh). 2025 Jun 23;12(32):e04717. doi: 10.1002/advs.202504717 (PMC12407315; doi:10.1002/advs.202504717)
Supplement: Supplementary file 1 — Supporting Information [file ADVS-12-e04717-s001.docx]

**Supplementary Information**

**An in-depth Study of the Solid Electrolyte Interphase Compositional Evolution in Sodium-Ion Batteries: Unravelling the Effects of a Na Metal Counter Electrode on the SEI**

Jack R. Fitzpatrick^1,2^, Beth E. Murdock^1,2^, Pardeep K. Thakur^3^, Tien-Lin Lee^3^, Sarah Fearn^4^, Andrew J. Naylor^5^, Deepnarayan Biswas^3^, Nuria Tapia-Ruiz^1,2,^*

^1^Department of Chemistry, Molecular Sciences Research Hub, Imperial College London, White City Campus, London, W12 0BZ, UK

^2^The Faraday Institution, Quad One, Harwell Science and Innovation Campus, Didcot, OX11 0RA, UK

^3^Diamond Light Source Ltd., Harwell Science and Innovation Campus, Didcot, Oxfordshire, OX11 0DE, United Kingdom

^4^Department of Materials, Imperial College London, Exhibition Road, London SW7 2AZ, UK

^5^Department of Chemistry–Ångström Laboratory, Uppsala University, Box 538, 751 21 Uppsala, Sweden

*Corresponding author: [n.tapia-ruiz@imperial.ac.uk](mailto:n.tapia-ruiz@imperial.ac.uk)

**Table of Contents**

[1. Notes 2](#_Toc189240532)

[1.1. Choice of electrochemical cycling protocols and cell setups used 2](#_Toc189240533)

[1.2. Spectral fitting of HAXPES data 3](#_Toc189240534)

[2. List of Tables 5](#_Toc189240535)

[3. List of Figures 9](#_Toc189240536)

[4. References 16](#_Toc189240537)

# **Notes**

## **Choice of electrochemical cycling protocols and cell setups used**

To conduct a fair comparison between a half-cell and a full-cell to elucidate the effects of a Na metal CE on the hard carbon SEI, the following conditions must be met for the full-cell: 1) The hard carbon electrode in the full-cell must experience a similar potential (i.e., the same degree of (de)sodiation) as the half-cell; and 2) the cathode must have enough Na inventory to account for SEI formation and reformation during cycling of the cell to prevent the electrolyte from acting as a Na^+^ ion reservoir. To meet these two requirements, a three-electrode full-cell with an oversized cathode was used in this study. A three-electrode cell was used to control the hard carbon voltage, see Figures S1a,b b for schematics of the half-cell and full-cell setups used. The full-cell was cycled by controlling the potential of the hard carbon anode vs. a Na metal ring reference in the voltage range of 2‒0.01 V, see Figures S1c,d d for the load curves of the cathode vs. the Na ref and the overall cell voltage. The cathode-to-anode mass ratio used for all of the full-cells was in the range of 2.031‒2.048, which equates to a cathode:anode reversible capacity ratio of ≈ 1.60.

It is important to note that the approaches described above have certain limitations and caveats. Firstly, Na is not excluded from the full-cell setup since a Na metal ring reference was used. The inherent instability of Na metal with carbonate-based electrolytes, as well as the creation of an unstable passivation layer on the Na metal, makes the redox potential of Na metal unstable.^1–3^ As a result, Na metal is an unreliable reference electrode. However, for the three-electrode PAT cell used in this study, a more stable reference electrode was not easily available without significant modifications to the cell setup, as the supplier only provided limited alternatives in the ring format used here, namely Li, Mg, and Na metals, activated carbon, and aluminum.^4^ Activated carbon was not used as the reference electrode because it has been demonstrated in the literature to be an unreliable reference electrode in organic systems due to reactions occurring between the carbon's surface functional groups and the electrolyte.^2^ For example, an activated carbon reference electrode exhibited a potential drift of 236 mV after cycling a glassy carbon working electrode in 1 M NaClO_4_ in PC.^2^ Aluminium was not used as the reference electrode because it forms an unstable passivation layer at high potentials and can dissolve into the electrolyte.^5–7^ Furthermore, Li and Mg metals were not used here to avoid any interference of their ionic forms in the SEI composition. Consequently, although Na metal is unreliable as a reference electrode,^1–3^ it is, in this instance, the simplest option for this commercial cell. We do acknowledge however, that in alternative cell setups, which provide more flexibility for the inclusion of different reference electrodes, such as a three-electrode Swagelok cell, a more stable, and as such, viable reference electrode could have been employed and should be employed in any future work on this topic. For example, a Na_3_V_2_(PO_4_)_3_ (NVP) electrode at a 50 % state of charge can be used as a stable reference electrode due to its long voltage plateau at ≈ 3.4 V.^8^ In addition it has been reported that a Ag/AgS_2_ quasi-reference electrode (QRE) in wire form, provides a stable potential in a lithium electrolyte systems, specifically, LiPF_6_ and LiTFSI in EC:DMC electrolyte and as such could be explored as a viable and more stable alternative to Na.^9^

In regard to still having Na metal present in the full-cell setup, in contrast to the Na metal CE in the half-cell, which is under pressure and faces the hard carbon anode directly, the ring format of the Na metal reference in the full-cell, which runs around the extremities of a separator, does not directly face the hard carbon electrode nor is it under any pressure, and the amount of Na present is much smaller than in the half-cell (see Figure S1b). Therefore, the ability of any formed species at the surface of the Na metal reference to influence SEI formation at the hard carbon anode is hindered compared to the Na metal CE in the half-cell.

Finally, we believe that the difference in cell setup (i.e., coin cell vs. PAT cell) has minimal effects on the formation and resultant stability of the SEI at the hard carbon electrode. The slightly lower specific capacity observed in the half-cell data compared to the full-cell data may be attributed to an increased polarization effect caused by the large interfacial resistance of the Na metal CE, which limits the kinetics of the cell and results in an early cut-off potential of the hard carbon electrode during sodiation. This phenomenon has been observed in sodium half-cells and represents another issue associated with using half-cells to gauge the performance of hard carbon electrodes.^10,11^ Furthermore, this discrepancy could also be due to differences in the pressure applied to the electrodes resulting from the distinct natures of the setups.^12,13^

## **Spectral fitting of HAXPES data**

A Shirley background was used to fit the HAXPES data, and all the components were fitted using the Gaussian/Lorentzian product function GL(30), which corresponds to a 30% Lorentzian and 70% Gaussian peak shape. The sp^2^ C peak in the C 1s spectra was fitted using a Lorentzian asymmetric peak shape LA(α, β, m) (α= 1.5, β= 2.5, m= 5; where α and β define the spread of the tail on either side of the Lorentzian component and m is the width of the Gaussian component). These line shapes were chosen following the work by Biesinger et al., who provided general fitting parameters for graphitic-type materials.^14^ Binding energy calibration was performed by setting the binding energy (BE) of the sp^3^ C peak in the C 1s spectra to 285.0 eV.

The HAXPES spectra presented in this work have been background corrected (i.e., the background has been subtracted), and the intensity of the spectra has been normalized against the total area above the background. Consequently, any differences in intensity shown between spectra are relative rather than absolute. This approach was done for two reasons: 1) to aid visual comparison of the spectra recorded at different energies since the measured intensity of photoelectron lines differs with varying X-ray energy, and 2) to ensure that differences in absolute intensity due to SEI heterogeneity from sample to sample were not considered in the analysis and subsequent discussion of the data.

The C 1s spectrum of the pristine sample was initially fitted using peak positions and full-width half-maxima (FWHM) as outlined in the work by Biesinger et al. for graphitic-type materials.^14^ To enhance the fit, a degree of variability in the previously defined peak positions and FWHM was permitted, as described in the literature.^15,16^ Specifically, peak FWHM was allowed to vary by ± 0.15 eV, resulting in an interval of 1.2‒1.5 eV, and peak positions were allowed to vary by ± 0.1 eV. The aforementioned applies to all peaks, except for the sp^2^ C peak (due to its different line shape), where the FWHM were allowed to vary between 0.8 and 1.0 eV. The π-π^*^ shake-up satellite peak is present due to the bonding-to-antibonding transition in aromatic species. Therefore, if the sp^2^ C is present, the π-π^*^ will also be present; thus, its position and intensity are linked relative to the sp^2^ C component in the fitted data.^14^

The C 1s spectra of electrodes at different states of charge exhibited additional peaks, absent in the pristine sample, necessitating a slightly modified fitting model compared to the one used for the pristine samples. This model was consistently applied to all these samples. The model was developed by initially fitting the spectrum of one of the samples (1^st^ sodiated sample in a half-cell) to the minimum number of peaks required to achieve an adequate fit (residual StD of the fit < 2). The 1^st^ sodiated sample in the half-cell was selected as it contained all the species present in all other samples. Peaks and their initial positions were determined based on standard reference databases^17,18^ and previous literature on the SEI in battery systems.^19–27^ The FWHM of all the peaks was constrained to vary between 1.2 and 1.5 eV, as previously defined for the pristine sample. This model was subsequently applied to all samples, with peak positions allowed to vary by ± 0.1 eV from the positions set for the 1^st^ sodiated sample in the half-cell.

A similar methodology was used to fit the O 1s and F 1s spectra of the samples, with the primary difference being slight variations in the interval over which the FWHM of the peaks were permitted to vary. In the O 1s spectra, the FWHM of the peaks were allowed to vary between 1.4 and 1.7 eV. In the F 1s spectra, the FWHM of the peak assigned to Na–F was constrained to vary between 1.2 and 1.5, while the peak assigned to NaPF_6_ and its subsequent decomposition products, e.g., Na_x_PF_y_O_z_, were allowed to vary over a broader interval of 1‒2.5 eV. A wider interval was selected in this case, as only one peak was used to describe multiple different species appearing at similar binding energies.

A Monte Carlo error analysis of the fitting models used in this work was carried out with the Casa XPS software.^15,28,29^ This analysis was performed to estimate the errors in the fitted peak areas based on the expected experimental noise/random noise in photoelectron spectroscopy data. The Monte Carlo analysis involved removing noise from the data assuming Poisson statistics, followed by the addition of random noise to the spectra to produce a set of simulated experimental results. In this study, 200 iterations were performed, generating 200 spectra for each sample with randomly added noise. The peak model created for that sample was then fitted to each newly generated spectrum. In this work, the errors in the percentage peak area contributions of the fitting components are reported as ± 2σ, where σ is the standard deviation calculated from the set of Monte Carlo simulations.

# **List of Tables**

Table S1. Peak positions, FWHMs, normalized areas, and peak assignments used to fit the C 1s, O 1s, and Na 1s spectra of the pristine hard carbon electrode sample at 2350 eV.

| Core Level  (Residual StD of fit) | Pristine hard carbon | | | Peak Assignment |
| --- | --- | --- | --- | --- |
|  | Binding Energy / eV | FWHM / eV | Normalized Area / % |  |
| C 1s  (1.33) | 284.2 | 1.0 | 58.9 ± 1.8 | sp^2^ C |
|  | 285.0 | 1.3 | 28.2 ± 1.5 | sp^3^ C |
|  | 286.9 | 1.2 | 6.5 ± 0.6 | C–O |
|  | 288.1 | 1.2 | 1.6 ± 0.6 | C=O |
|  | 288.7 | 1.2 | 1.1 ± 0.5 | O–C=O |
|  | 290.7 | 2.7 | 3.6 ± 0.1 | π–π* |
| O 1s  (1.05) | 530.3 | 1.7 | 5.5 ± 1.9 | R–O–Na/Na_2_O |
|  | 531.6 | 1.4 | 21.4 ± 2.0 | C=O/O–C=O/CO_3_^2-^ |
|  | 533.2 | 1.6 | 73.1 ± 2.2 | C–O |
| Na 1s | 1071.4 |  | | Inorganic and organic Na compounds |

Table S2. Peak positions, FWHMs, normalized areas, and peak assignments to fit the C 1s, O 1s, F 1s, and Na 1s spectra of the hard carbon half-cell (HC) electrode samples measured at 2350 eV.

| Core Level | HC OCV | | | HC 1^st^ Sodiation | | | HC 2^nd^ Sodiation | | | HC 10^th^ Sodiation | | | Peak Assignment |
| --- | --- | --- | --- | --- | --- | --- | --- | --- | --- | --- | --- | --- | --- |
|  | Binding Energy / eV | FWHM / eV | Normalized Area / % | Binding Energy / eV | FWHM / eV | Normalized Area / % | Binding Energy / eV | FWHM / eV | Normalized Area / % | Binding Energy / eV | FWHM / eV | Normalized Area / % |  |
| C 1s | 283.9 | 1.0 | 26.9 ± 0.7 | 283.3 | 1.0 | 5.8 ± 1.2 | 283.2 | 0.8 | 3.6 ± 0.8 | 283.4 | 1.0 | 3.1 ± 0.8 | sp^2^ C |
|  | 285.0 | 1.2 | 49.8 ± 0.9 | 285.0 | 1.2 | 63.1 ± 1.7 | 285.0 | 1.5 | 55.8 ± 1.5 | 285.0 | 1.2 | 51.7 ± 1.4 | sp^3^ C |
|  | 286.9 | 1.5 | 13.9 ± 0.4 | 286.9 | 1.5 | 11.8 ± 0.8 | 286.8 | 1.5 | 15.2 ± 0.7 | 286.7 | 1.4 | 17.8 ± 1.0 | C–O |
|  | 288.7 | 1.5 | 3.6 ± 0.3 | 288.7 | 1.5 | 5.1 ± 1.3 | 288.9 | 1.3 | 12.8 ± 1.6 | 288.7 | 1.2 | 12.3 ± 0.9 | O–C=O |
|  | 290.2 | 1.5 | 4.1 ± 0.3 | 289.7 | 1.5 | 13.9 ± 1.2 | 289.9 | 1.5 | 12.4 ± 1.2 | 289.9 | 1.5 | 14.8 ± 0.7 | CO_3_^2-^ |
|  | 290.4 | 2.7 | 1.6 ± 0.0 | 290.4 | 2.7 | 0.4 ± 0.1 | 289.7 | 2.7 | 0.2 ± 0.1 | 289.9 | 1.5 | 0.2 ± 0.1 | π–π* |
| O 1s | 530.4 | 1.7 | 2.6 ± 0.7 | 530.4 | 1.7 | 1.6 ± 1.3 | 530.5 | 1.7 | 12.9 ± 3.5 | 530.5 | 1.7 | 10.7 ± 4.4 | R–O–Na/Na_2_O |
|  | 531.7 | 1.7 | 58.2 ± 0.8 | 531.6 | 1.7 | 78.4 ± 1.9 | 531.6 | 1.7 | 73.2 ± 4.2 | 531.5 | 1.6 | 67.5 ± 4.6 | C=O/O–C=O/CO_3_^2-^ |
|  | 533.4 | 1.7 | 39.3 ± 0.6 | 533.5 | 1.7 | 20.0 ± 1.2 | 533.5 | 1.6 | 13.9 ± 1.2 | 533.4 | 1.7 | 21.8 ± 1.0 | C–O |
| F 1s | 684.4 | 1.4 | 23.8 ± 0.4 | 684.5 | 1.3 | 78.9 ± 0.3 | 684.4 | 1.3 | 76.9 ± 0.5 | 684.0 | 1.4 | 23.5 ± 1.3 | Na–F |
|  | 687.8 | 1.7 | 76.2 ± 0.4 | 687.7 | 1.8 | 21.2 ± 0.3 | 687.5 | 1.8 | 23.1 ± 0.5 | 687.4 | 1.8 | 76.5 ± 1.3 | Na_x_PF_y_ / Na_x_PF_y_O_z_ |
| Na 1s | 1072.1 |  | | 1071.7 |  | | 1071.5 |  | | 1071.4 |  | | Inorganic and organic Na compounds |

Table S3. Peak positions, FWHMs, normalized areas, and peak assignments to fit the C 1s, O 1s, F 1s, and Na 1s spectra of the hard carbon full-cell (FC) electrode samples measured at 2350 eV.

| Core Level | FC OCV | | | FC 1^st^ Sodiation | | | FC 2^nd^ Sodiation | | | FC 10^th^ Sodiation | | | Peak Assignment |
| --- | --- | --- | --- | --- | --- | --- | --- | --- | --- | --- | --- | --- | --- |
|  | Binding Energy / eV | FWHM / eV | Normalized Area / % | Binding Energy / eV | FWHM / eV | Normalized Area / % | Binding Energy / eV | FWHM / eV | Normalized Area / % | Binding Energy / eV | FWHM / eV | Normalized Area / % |  |
| C 1s | 284.4 | 1.0 | 49.7 ± 2.3 | 283.4 | 1.0 | 8.2 ± 1.2 | 283.3 | 0.9 | 3.7 ± 1.3 | 283.2 | 0.8 | 1.4 ± 0.4 | sp^2^ C |
|  | 285.0 | 1.2 | 35.2 ± 2.4 | 285.0 | 1.4 | 59.9 ± 2.0 | 285.0 | 1.4 | 60.5 ± 2.5 | 285.0 | 1.2 | 72.7 ± 1.0 | sp^3^ C |
|  | 286.9 | 1.5 | 8.7 ± 0.9 | 286.7 | 1.4 | 12.1 ± 1.3 | 286.7 | 1.5 | 12.3 ± 1.4 | 286.7 | 1.5 | 9.8 ± 0.7 | C–O |
|  | 287.9 | 1.5 | 1.4 ± 0.6 | N/A | | | | | | | | | C=O |
|  | 288.9 | 1.2 | 1.6 ± 0.3 | 288.9 | 1.4 | 12.1 ± 2.4 | 288.9 | 1.5 | 15.9 ± 1.7 | 288.8 | 1.3 | 10.4 ± 1.2 | O–C=O |
|  | 289.9 | 1.2 | 0.4 ± 0.4 | 289.8 | 1.3 | 7.3 ± 1.8 | 289.8 | 1.4 | 7.4 ± 2.0 | 289.9 | 1.5 | 5.7 ± 1.2 | CO_3_^2-^ |
|  | 290.9 | 2.7 | 3.0 ± 0.1 | 289.8 | 2.7 | 0.5 ± 0.1 | 289.8 | 2.7 | 0.2 ± 0.1 | 289.7 | 2.7 | 0.1 ± 0.0 | π–π* |
| O 1s | 530.3 | 1.4 | 2.0 ± 1.1 | 530.4 | 1.5 | 21.6 ± 3.6 | 530.5 | 1.4 | 29.8 ± 2.7 | 530.4 | 1.4 | 8.6 ± 1.3 | R–O–Na/Na_2_O |
|  | 531.7 | 1.4 | 19.1 ± 1.0 | 531.6 | 1.6 | 61.2 ± 4.6 | 531.7 | 1.5 | 52.5 ± 3.3 | 531.7 | 1.6 | 69.1 ± 2.2 | C=O/O–C=O/CO_3_^2-^ |
|  | 533.4 | 1.7 | 79.0 ± 1.3 | 533.4 | 1.7 | 17.2 ± 1.4 | 533.4 | 1.7 | 17.7 ± 1.2 | 533.5 | 1.7 | 22.4 ± 1.0 | C–O |
| F 1s |  |  | 0.0 ± 0.0 | 684.4 | 1.2 | 79.5 ± 0.3 | 684.4 | 1.2 | 63.1 ± 0.2 | 684.5 | 1.3 | 61.6 ± 0.3 | Na–F |
|  | 687.8 | 2.5 | 100.0 ± 0.0 | 687.5 | 1.8 | 20.5 ± 0.3 | 687.7 | 1.7 | 36.9 ± 0.2 | 687.7 | 1.7 | 38.3 ± 0.3 | Na_x_PF_y_ / Na_x_PF_y_O_z_ |
| Na 1s | 1072.2/ 1074.0 |  | | 1071.4 |  | | 1071.5 |  | | 1071.7 |  | | Inorganic and organic Na compounds |

Table S4. Residual standard deviations of the C 1s, O 1s, and F 1s spectra fittings for all samples measured at 2350 eV.

| Core Level | Residual StD of Fit | | | | | | | | |
| --- | --- | --- | --- | --- | --- | --- | --- | --- | --- |
|  | Pristine | HC OCV | HC 1^st^ Sodiation | HC 2^nd^ Sodiation | HC 10^th^ Sodiation | FC OCV | FC 1^st^ Sodiation | FC 2^nd^ Sodiation | FC 10^th^ Sodiation |
| C 1s | 1.33 | 1.50 | 1.05 | 1.07 | 1.14 | 1.20 | 1.01 | 0.98 | 1.12 |
| O 1s | 1.05 | 1.57 | 1.01 | 1.02 | 1.08 | 1.30 | 0.96 | 1.15 | 1.17 |
| F 1s |  | 1.10 | 1.52 | 1.07 | 1.28 | 1.26 | 1.72 | 1.42 | 1.25 |

Table S5. Peak positions, FWHMs, normalized areas, and peak assignments to fit the C 1s, O 1s, F 1s, and Na 1s spectra of the pristine hard carbon electrode sample measured at 7050 eV.

| Core Level | Pristine | | | Peak Assignment |
| --- | --- | --- | --- | --- |
|  | Binding Energy / eV | FWHM / eV | Normalized Area / % |  |
| C 1s | 284.2 | 1.0 | 62.3 ± 1.8 | sp^2^ C |
|  | 285.0 | 1.5 | 25.0 ± 1.9 | sp^3^ C |
|  | 286.7 | 1.3 | 5.0 ± 0.9 | C–O |
|  | 288.1 | 1.2 | 1.6 ± 0.7 | C=O |
|  | 288.9 | 1.5 | 2.3 ± 0.9 | O–C=O |
|  | 290.6 | 2.7 | 3.8 ± 0.1 | π–π* |
| O 1s | 530.3 | 1.7 | 10.4 ± 2.5 | R–O–Na/Na_2_O |
|  | 531.6 | 1.4 | 22.9 ± 3.8 | C=O/O–C=O/CO_3_^2-^ |
|  | 533.2 | 1.6 | 66.7 ± 3.5 | C–O |
| Na 1s | 1071.7 |  |  | Inorganic and organic Na compounds |

Table S6. Peak positions, FWHMs, normalized areas, and peak assignments to fit the C 1s, O 1s, F 1s, and Na 1s spectra of the hard carbon half-cell (HC) electrode samples measured at 7050 eV.

| Core Level | HC OCV | | | HC 1^st^ Sodiation | | | HC 2^nd^ Sodiation | | | HC 10^th^ Sodiation | | | Peak Assignment |
| --- | --- | --- | --- | --- | --- | --- | --- | --- | --- | --- | --- | --- | --- |
|  | Binding Energy / eV | FWHM / eV | Normalized Area / % | Binding Energy / eV | FWHM / eV | Normalized Area / % | Binding Energy / eV | FWHM / eV | Normalized Area / % | Binding Energy / eV | FWHM / eV | Normalized Area / % |  |
| C 1s | 284.0 | 1.0 | 48.7 ± 1.2 | 283.5 | 1.0 | 22.8 ± 1.4 | 283.5 | 1.0 | 18.9 ± 0.9 | 283.3 | 1.0 | 12.0 ± 0.8 | sp^2^ C |
|  | 285.0 | 1.3 | 32.4 ± 1.1 | 285.0 | 1.5 | 46.0 ± 1.2 | 285.0 | 1.5 | 46.5 ± 1.2 | 285.0 | 1.4 | 44.6 ± 1.3 | sp^3^ C |
|  | 286.9 | 1.5 | 10.2 ± 0.4 | 286.9 | 1.5 | 11.7 ± 0.7 | 286.7 | 1.5 | 15.2 ± 0.8 | 286.8 | 1.5 | 16.7 ± 0.6 | C–O |
|  |  |  | 0.0 ± 0.0 |  | | 0.0 ± 0.0 |  |  | 0.0 ± 0.0 |  |  | 0.0 ± 0.0 | C=O |
|  | 288.7 | 1.5 | 3.2 ± 0.4 | 288.7 | 1.5 | 5.6 ± 1.1 | 288.9 | 1.5 | 8.1 ± 1.1 | 288.8 | 1.2 | 11.4 ± 0.7 | O–C=O |
|  | 290.2 | 1.5 | 2.6 ± 0.4 | 289.8 | 1.5 | 12.6 ± 1.6 | 289.9 | 1.2 | 10.2 ± 0.9 | 289.9 | 1.5 | 14.6 ± 0.7 | CO_3_^2-^ |
|  | 290.5 | 2.7 | 3.0 ± 0.1 | 290.0 | 2.7 | 1.4, ± 0.1 | 289.9 | 2.7 | 1.1 ± 0.1 | 289.8 | 2.7 | 0.7 ± 0.1 | π–π* |
| O 1s | 530.4 | 1.4 | 4.9 ± 0.9 | 530.4 | 1.6 | 11.1 ± 1.0 | 530.5 | 1.7 | 21.0 ± 1.5 | 530.6 | 1.7 | 20.7 ± 4.8 | R–O–Na/Na_2_O |
|  | 531.8 | 1.6 | 47.5 ± 1.8 | 531.7 | 1.7 | 68.8 ± 1.2 | 531.7 | 1.7 | 62.8 ± 1.9 | 531.6 | 1.6 | 62.8 ± 5.3 | C=O/O–C=O/CO_3_^2-^ |
|  | 533.4 | 1.7 | 47.6 ± 1.1 | 533.5 | 1.7 | 20.2 ± 0.6 | 533.5 | 1.7 | 16.2 ± 0.6 | 533.5 | 1.7 | 16.5 ± 1.0 | C–O |
| F 1s | 684.4 | 1.4 | 24.3 ± 0.7 | 684.5 | 1.3 | 78.9 ± 0.5 | 684.4 | 1.3 | 58.9 ± 0.8 | 684.1 | 1.3 | 20.5 ± 0.7 | Na–F |
|  | 687.7 | 1.7 | 75.7 ± 0.7 | 687.6 | 1.8 | 21.1 ± 0.5 | 687.6 | 1.8 | 41.1 ± 0.8 | 687.8 | 1.7 | 79.5 ± 0.7 | Na_x_PF_y_ / Na_x_PF_y_O_z_ |
| Na 1s | 1071.8 |  |  | 1071.6 |  |  | 1071.5 |  |  | 1071.3 |  |  | Inorganic and organic Na compounds |

Table S7. Peak positions, FWHMs, normalized areas, and peak assignments to fit the C 1s, O 1s, F 1s, and Na 1 s spectra of the hard carbon full-cell (FC) electrode samples measured at 7050 eV.

| Core Level | FC OCV | | | FC 1^st^ Sodiation | | | FC 2^nd^ Sodiation | | | FC 10^th^ Sodiation | | | Peak Assignment |
| --- | --- | --- | --- | --- | --- | --- | --- | --- | --- | --- | --- | --- | --- |
|  | Binding Energy / eV | FWHM / eV | Normalized Area / % | Binding Energy / eV | FWHM / eV | Normalized Area / % | Binding Energy / eV | FWHM / eV | Normalized Area / % | Binding Energy / eV | FWHM / eV | Normalized Area / % |  |
| C 1s | 284.3 | 1.0 | 56.3 ± 2.6 | 283.4 | 1.0 | 25.9 ± 0.8 | 283.4 | 1.0 | 22.2 ± 1.2 | 283.6 | 1.0 | 15.5 ± 1.5 | sp^2^ C |
|  | 285.0 | 1.5 | 28.7 ± 2.5 | 285.0 | 1.5 | 46.1 ± 1.3 | 285.0 | 1.5 | 48.9 ± 1.6 | 285.0 | 1.3 | 55.5 ± 2.6 | sp^3^ C |
|  | 286.9 | 1.4 | 6.6 ± 1.4 | 286.7 | 1.5 | 10.7 ± 0.8 | 286.7 | 1.5 | 9.2 ± 1.1 | 286.7 | 1.5 | 11.0 ± 1.0 | C–O |
|  | 287.9 | 1.5 | 2.5 ± 1.0 |  |  | 0.0 ± 0.0 |  |  | 0.0 ± 0.0 |  |  | 0.0 ± 0.0 | C=O |
|  | 288.8 | 1.2 | 1.1 ± 0.7 | 288.9 | 1.5 | 5.3 ± 1.1 | 288.9 | 1.5 | 6.8 ± 1.4 | 288.9 | 1.4 | 8.1 ± 1.5 | O–C=O |
|  | 289.9 | 1.5 | 1.3 ± 0.6 | 289.8 | 1.5 | 10.5 ± 1.1 | 289.7 | 1.5 | 11.6 ± 1.4 | 289.8 | 1.5 | 8.9 ± 1.4 | CO₃²⁻ |
|  | 290.8 | 2.7 | 3.4 ± 0.2 | 289.9 | 2.7 | 1.6 ± 0.1 | 289.9 | 2.7 | 1.3 ± 0.1 | 290.0 | 2.7 | 0.9 ± 0.1 | π–π* |
| O 1s | 530.3 | 1.7 | 4.9 ± 1.8 | 530.5 | 1.4 | 25.0 ± 1.0 | 530.5 | 1.4 | 30.9 ± 2.2 | 530.4 | 1.4 | 16.4 ± 0.8 | R–O–Na/Na_2_O |
|  | 531.7 | 1.4 | 20.4 ± 1.3 | 531.7 | 1.7 | 64.7 ± 1.2 | 531.7 | 1.6 | 58.4 ± 2.6 | 531.7 | 1.7 | 68.7 ± 1.0 | C=O/O–C=O/CO_3_^2-^ |
|  | 533.4 | 1.7 | 74.7 ± 1.7 | 533.6 | 1.7 | 10.4 ± 0.7 | 533.5 | 1.6 | 10.7 ± 1.4 | 533.5 | 1.7 | 15.0 ± 0.7 | C–O |
| F 1s |  |  | 0.0 ± 0.0 | 684.6 | 1.3 | 79.9 ± 0.3 | 684.5 | 1.3 | 70.0 ± 0.4 | 684.6 | 1.3 | 67.0 ± 0.3 | Na–F |
|  | 688.1 | 2.5 | 100.0 ± 0.0 | 687.7 | 1.8 | 20.1 ± 0.3 | 687.8 | 1.7 | 30.1 ± 0.4 | 687.8 | 1.7 | 33.1 ± 0.3 | Na_x_PF_y_ / Na_x_PF_y_O_z_ |
| Na 1s | 1072.0/ 1073.8 |  | | 1071.5 |  | | 1071.5 |  | | 1071.8 |  | | Inorganic and organic Na compounds |

Table S8. Residual standard deviations of the C 1s, O 1s, and F 1s spectra fittings for all the samples measured at 7050 eV.

| Core Level | Residual StD of Fit | | | | | | | | | |
| --- | --- | --- | --- | --- | --- | --- | --- | --- | --- | --- |
|  | Pristine | HC OCV | HC 1^st^ Sodiation | HC 2^nd^ Sodiation | HC 10^th^ Sodiation | FC OCV | FC 1^st^ Sodiation | FC 2^nd^ Sodiation | FC 10^th^ Sodiation |  |
| C 1s | 1.37 | 1.21 | 1.11 | 1.32 | 1.32 | 1.20 | 1.31 | 1.10 | 1.05 |  |
| O 1s | 0.98 | 1.43 | 1.05 | 1.21 | 1.23 | 1.10 | 1.16 | 1.26 | 1.47 |  |
| F 1s | N/A | 1.15 | 1.27 | 1.01 | 1.51 | 1.39 | 1.31 | 1.42 | 1.12 |  |

Table S9. Calculated atomic concentrations (in %) and associated errors (2σ) from Monte Carlo simulations of all samples measured at 2350 and 7050 eV.

| Atomic Concentrations % - 2350 eV | | | | | | | | |
| --- | --- | --- | --- | --- | --- | --- | --- | --- |
| Sample | O 1s | Na 1s | C 1s - sp^2^ C | C 1s - Other | F 1s - NaF | F 1s - Other | P 2p | N 1s |
| Pristine | 7.8 ± 1.2 | 0.9 ± 0.3 | 55.4 ± 3.5 | 33.2 ± 3.3 | 0.0 ± 0.0 | 0.0 ± 0.0 | 0.2 ± 0.7 | 2.5 ± 1.8 |
| HC OCV | 18.0 ± 1.2 | 11.7 ± 0.6 | 15.8 ± 1.0 | 39.4 ± 1.8 | 2.8 ± 0.2 | 9.0 ± 0.7 | 1.9 ± 0.9 | 1.4 ± 1.5 |
| HC 1^st^ | 15.2 ± 1.1 | 35.4 ± 1.3 | 1.5 ± 0.6 | 23.7 ± 2.3 | 19 ± 0.9 | 5.1 ± 0.3 | 0.0 ± 0.0 | 0.0 ± 0.0 |
| HC 2^nd^ | 22.2 ± 1.1 | 28.6 ± 1.0 | 1.3 ± 0.5 | 32.1 ± 2.5 | 11.9 ± 0.8 | 3.6 ± 0.3 | 0.3 ± 1.5 | 0.0 ± 0.0 |
| HC 10^th^ | 35.8 ± 2.8 | 19.1 ± 1.5 | 1.2 ± 0.6 | 35.3 ± 3.6 | 1.4 ± 0.4 | 4.5 ± 1.2 | 2.8 ± 3.9 | 0.0 ± 0.0 |
| FC OCV | 8.3 ± 1.3 | 2.1 ± 0.3 | 43.5 ± 4.2 | 39.1 ± 4.6 | 0.0 ± 0.0 | 5.1 ± 0.9 | 1.1 ± 1.2 | 0.8 ± 3.3 |
| FC 1^st^ | 16.0 ± 0.9 | 35.7 ± 1.3 | 2.0 ± 0.6 | 21.0 ± 2.6 | 20.2 ± 0.9 | 5.2 ± 0.3 | 0.0 ± 2.0 | 0.0 ± 0.0 |
| FC 2^nd^ | 19.1 ± 1.2 | 30.6 ± 1.2 | 0.8 ± 0.5 | 19.7 ± 2.4 | 17.1 ± 0.8 | 10.0 ± 0.5 | 2.7 ± 2.1 | 0.0 ± 0.0 |
| FC 10^th^ | 16.4 ± 1.2 | 22.9 ± 1.0 | 0.6 ± 0.3 | 38.7 ± 2.6 | 13.1 ± 0.7 | 8.1 ± 0.5 | 0.3 ± 1.8 | 0.0 ± 0.0 |

| Atomic Concentrations % - 7050 eV | | | | | | | | |
| --- | --- | --- | --- | --- | --- | --- | --- | --- |
| Sample | O 1s | Na 1s | C 1s - sp^2^ C | C 1s - Other | F 1s - NaF | F 1s - Other | P 2p | N 1s |
| Pristine | 7.2 ± 1.3 | 0.8 ± 0.3 | 59.7 ± 3.7 | 30.7 ± 4.4 | 0.0 ± 0.0 | 0.0 ± 0.0 | 0.0 ± 0.0 | 1.7 ± 1.1 |
| HC OCV | 13.0 ± 1.1 | 9.4 ± 0.5 | 35.1 ± 1.9 | 32.8 ± 2.1 | 1.7 ± 0.2 | 5.2 ± 0.7 | 0.0 ± 0.0 | 2.8 ± 0.8 |
| HC 1^st^ | 19.3 ± 1.2 | 31.6 ± 1.5 | 7.2 ± 1.1 | 22.6 ± 2.4 | 14.3 ± 1.1 | 3.8 ± 0.3 | 0.0 ± 0.0 | 1.1 ± 1.4 |
| HC 2^nd^ | 25.9 ± 2.0 | 26.1 ± 1.7 | 7.2 ± 0.9 | 28.8 ± 3.0 | 6.4 ± 0.9 | 4.4 ± 0.7 | 0.7 ± 3.1 | 0.6 ± 1.7 |
| HC 10^th^ | 33.4 ± 1.7 | 22.3 ± 1.1 | 5.0 ± 0.7 | 34.1 ± 2.4 | 1.0 ± 0.2 | 4.0 ± 0.9 | 0.0 ± 0.0 | 0.2 ± 1.4 |
| FC OCV | 7.6 ± 1.3 | 1.4 ± 0.3 | 49.1 ± 4.6 | 33.1 ± 5.4 | 0.0 ± 0.0 | 4.9 ± 1.2 | 0.0 ± 0.0 | 3.9 ± 1.0 |
| FC 1^st^ | 18.2 ± 1.2 | 34.6 ± 1.5 | 7.3 ± 0.8 | 19.3 ± 2.1 | 16.3 ± 1.0 | 4.1 ± 0.3 | 0.0 ± 0.0 | 0.2 ± 1.3 |
| FC 2^nd^ | 18.4 ± 1.4 | 32.4 ± 1.6 | 5.8 ± 0.9 | 18.8 ± 2.4 | 14.6 ± 1.0 | 6.3 ± 0.4 | 2.6 ± 2.3 | 1.1 ± 1.4 |
| FC 10^th^ | 17.9 ± 1.2 | 31.1 ± 1.6 | 4.5 ± 0.9 | 22.7 ± 3.0 | 15.8 ± 1.0 | 7.8 ± 0.5 | 0.0 ± 0.0 | 0.3 ± 1.5 |

#
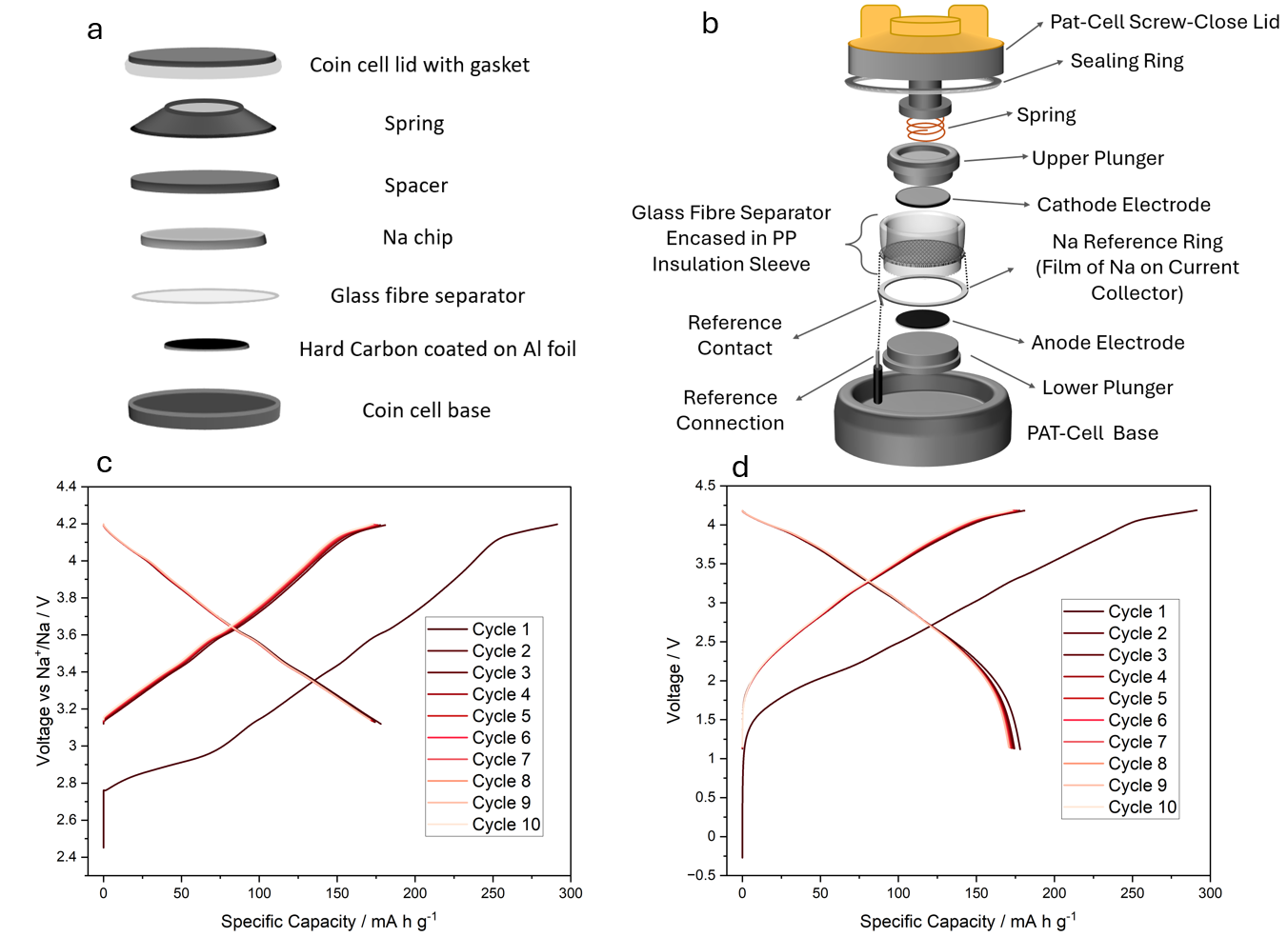
**List of Figures**

Figure S1 - Schematics of the cells used in this study. a) A two-electrode half-cell using a coin cell and b) a three-electrode full-cell using a PAT cell. Additional load curves from the three-electrode full-cell over the 10 cycles. c) Load curve of the cathode material vs the Na reference, and d) load curve of the cathode material vs the HC anode material, i.e., overall cell voltage.


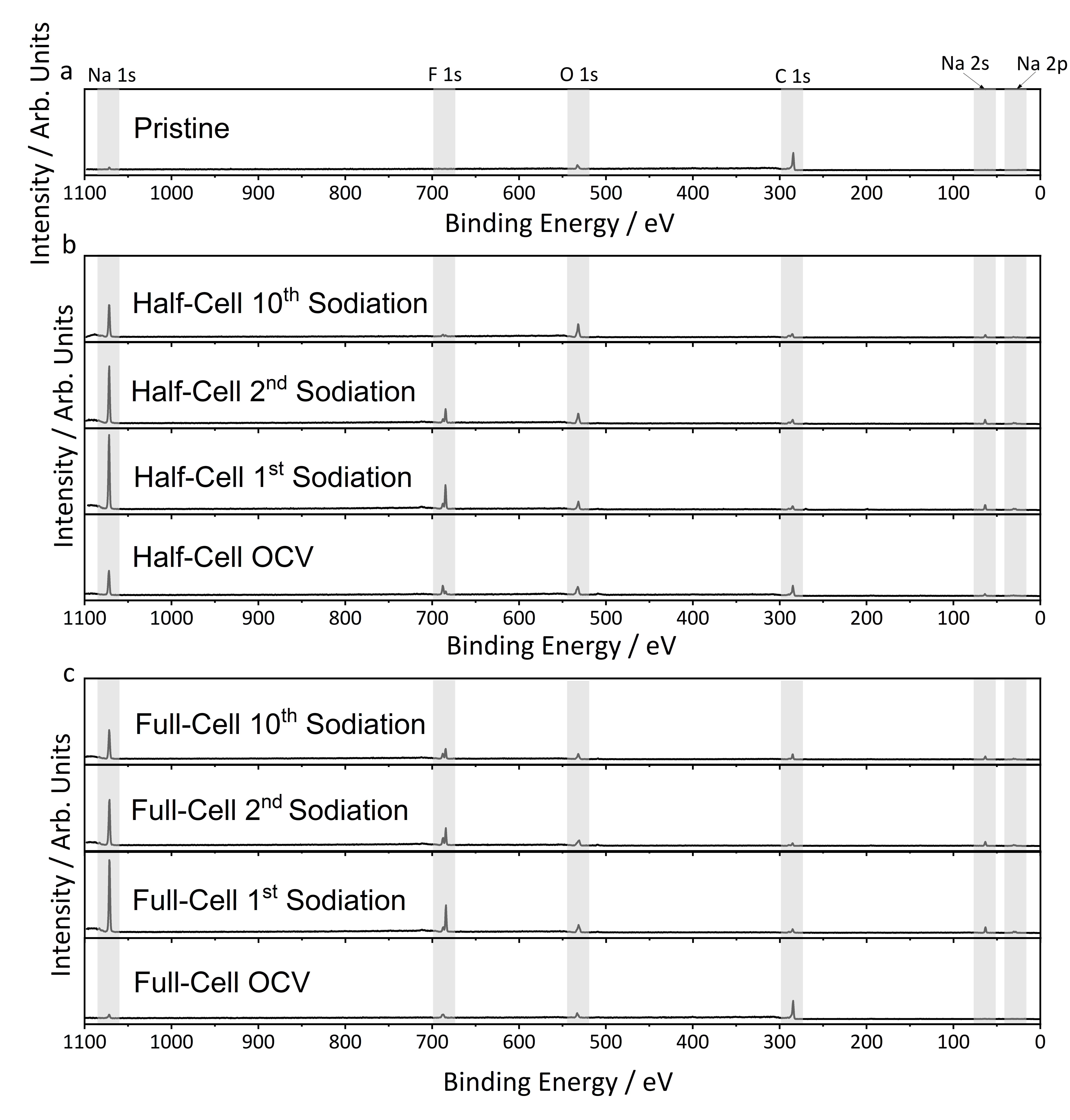
Figure S2. HAXPES survey scans obtained at 2350 eV for hard carbon electrodes in a) pristine state and extracted from b) half-cells and c) full-cells after a 10 h OCV rest period and the 1^st^, 2^nd^, and 10^th^ sodiation cycles. The Na 1s, F 1s, O 1s, C 1s, Na 2s, and Na 2p regions are all highlighted.


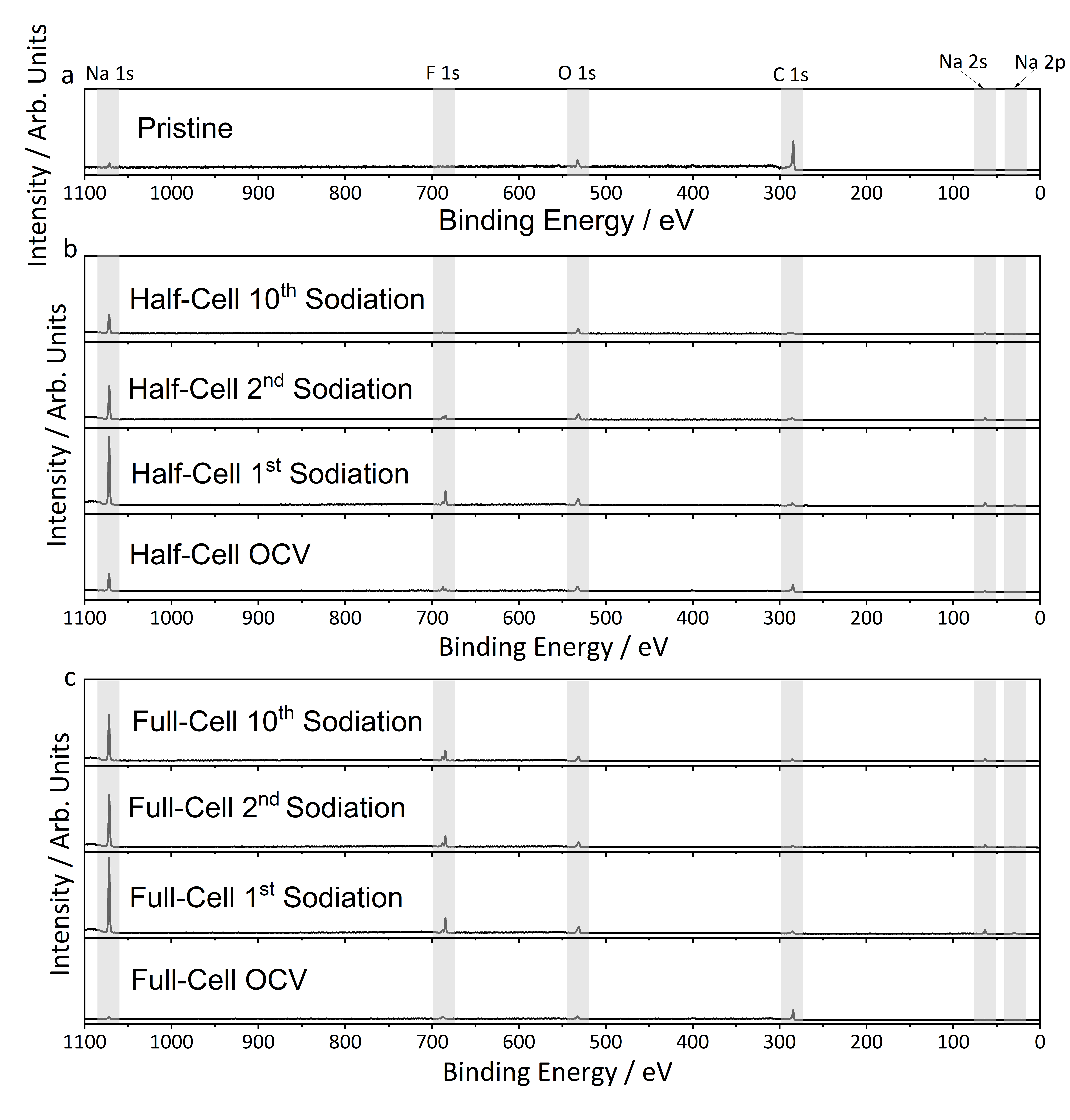
Figure S3. HAXPES survey scans obtained at 7050 eV for hard carbon electrodes in a) pristine state and extracted from b) half-cells and c) full-cells after a 10 h OCV rest period and the 1^st^, 2^nd^, and 10^th^ sodiation cycles. The Na 1s, F 1s, O 1s, C 1s, Na 2s, and Na 2p regions are all highlighted.

Figure S4. a) Na 1s and b) P 2p HAXPES spectra obtained at 2350 eV for hard carbon electrodes in the pristine state and extracted from half-cells and full-cells after a 10 h OCV rest period and the 1^st^, 2^nd^, and 10^th^ sodiation cycles. The dashed lines shown in a) are positioned at the center of the Na 1s peak for the half-cell and full-cell 1^st^ sodiation samples to emphasize the shift in the Na 1s peak as cycling progressed.


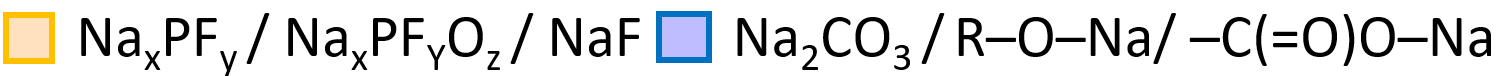

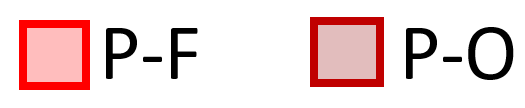

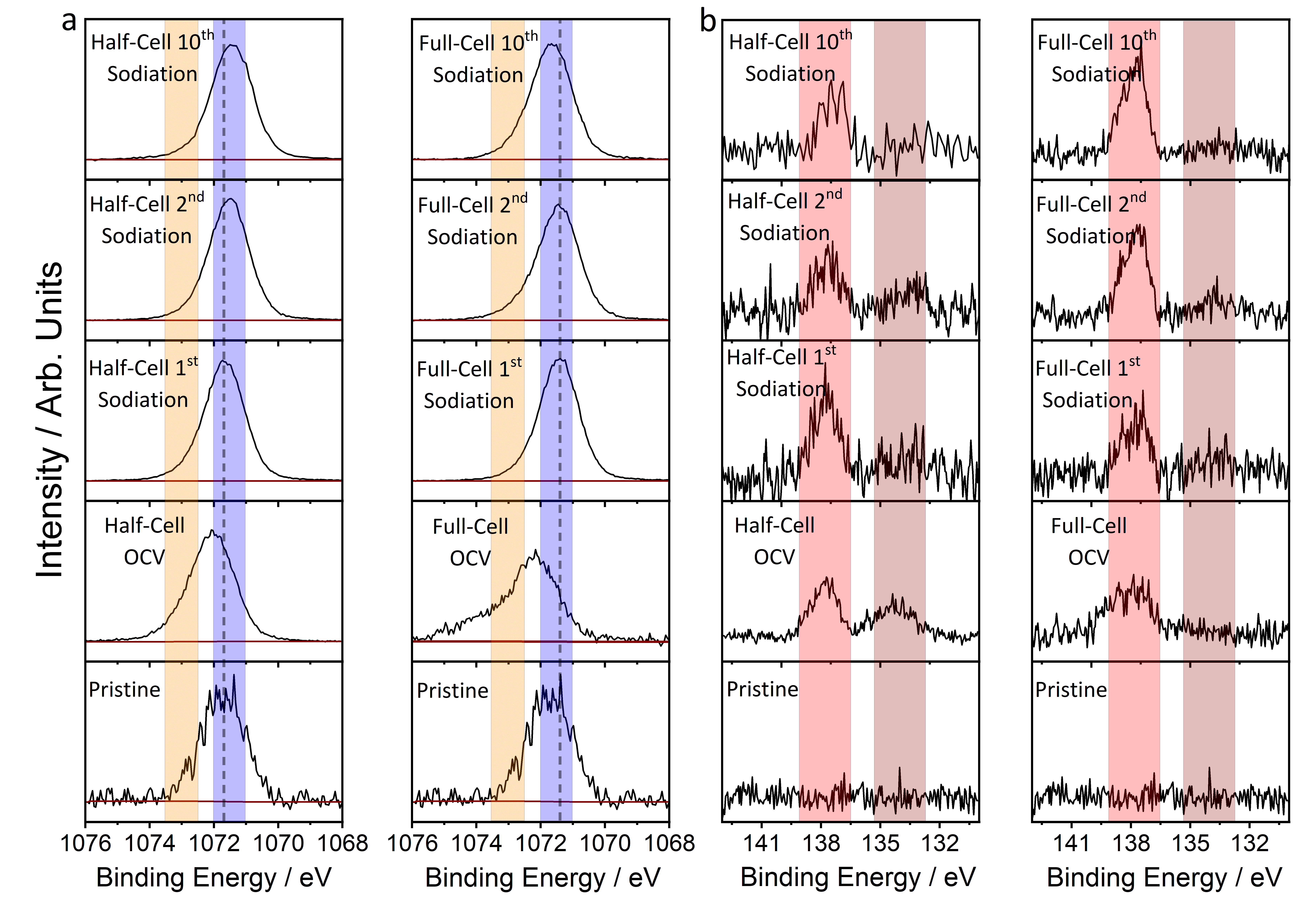



Figure S5. Ex-situ HAXPES data obtained at 7050 eV. a) C 1s, b) O1s, and c) F 1s spectra for hard carbon electrodes in the pristine state and extracted from half-cells (HC) and full-cells (FC) following a 10 h OCV rest period. d) Area percentage contributions of the sp^2^ C, sp^3^ C, and oxidized carbon species (C–O, C=O, O–C=O, and CO_3_^2-^) components to the C 1s spectra fittings. e) Calculated atomic concentrations of the elements/compounds (in at.%) and associated errors observed on the surface of the hard carbon electrodes in the pristine state and extracted from half-cells and full-cells after a 10 h OCV rest period. The plotted errors represent ± 2σ, where σ is the standard deviation in the fitted peak areas/atomic concentration calculated from the set of Monte Carlo simulations (refer to Note 1.2 in the SI).


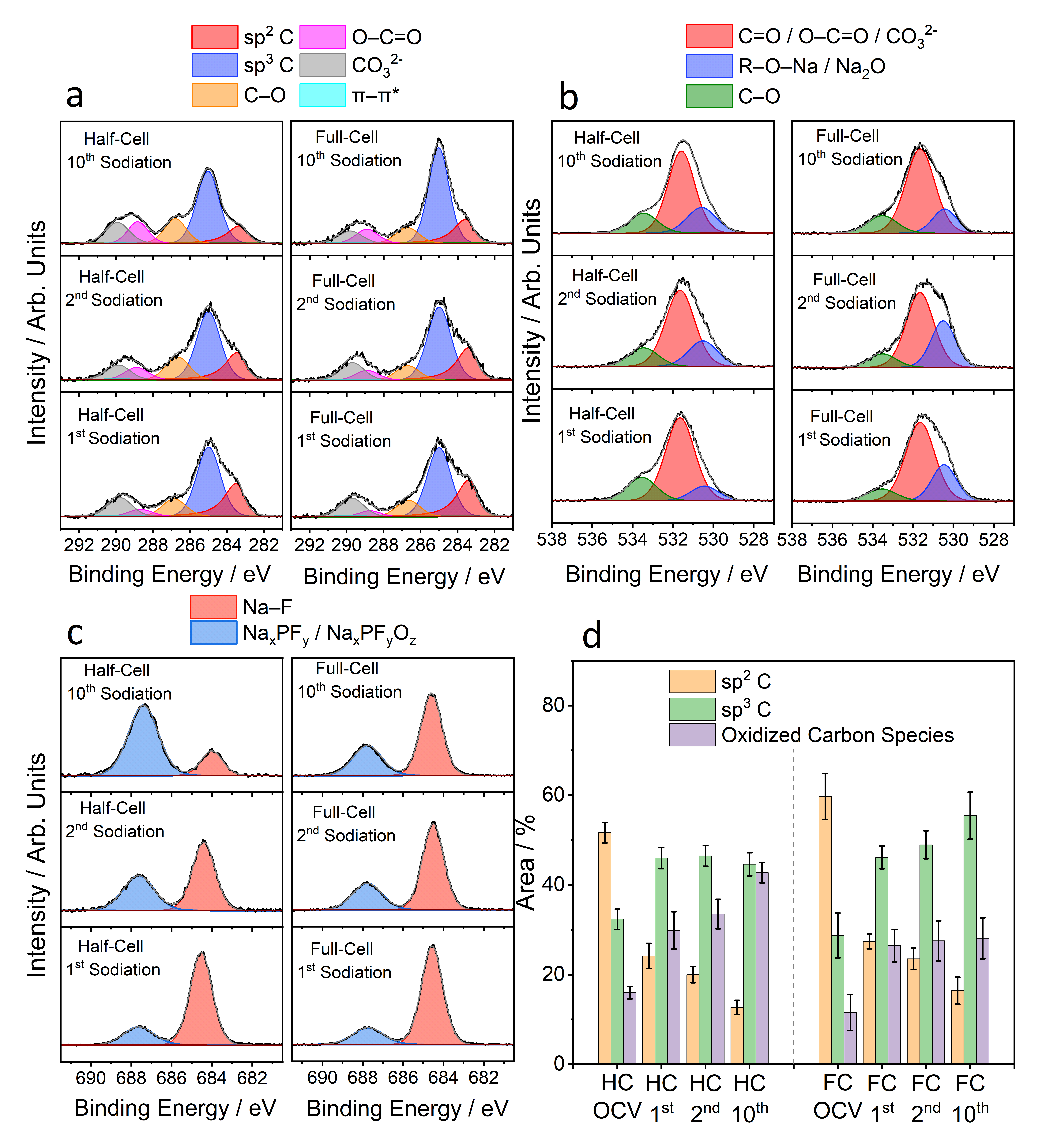
Figure S6. Ex-situ HAXPES data obtained at 7050 eV. a) C 1s, b) 1s, and c) F 1s spectra for hard carbon electrodes extracted from half-cells (HC) and full-cells (FC) after the 1^st^, 2^nd^, and 10^th^ sodiation cycles. d) Area percentage contributions of the sp^2^ C, sp^3^ C, and oxidized carbon species (C–O, C=O, O–C=O, and CO_3_^2-^) components to the C 1s spectra fittings. The plotted errors represent ± 2σ, where σ is the standard deviation in the fitted peak areas/atomic concentration calculated from the set of Monte Carlo simulations (refer to Note 1.2 in the SI).


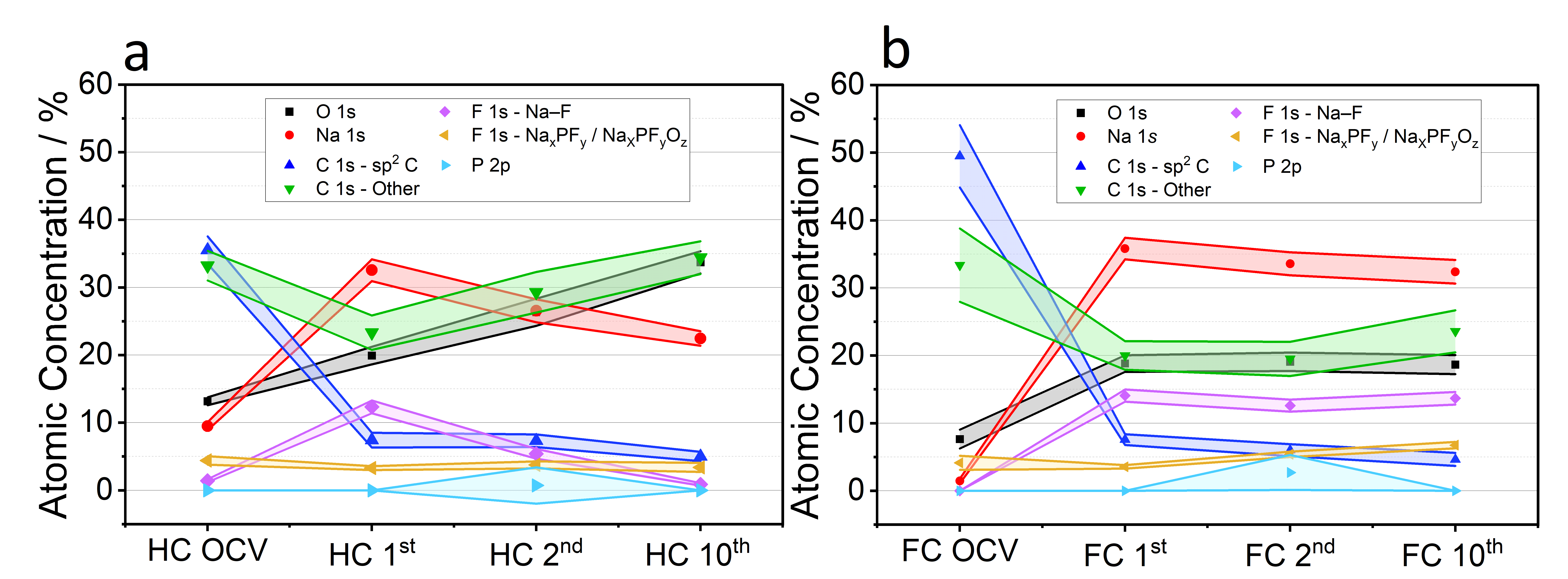

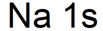


Figure S7. Calculated atomic concentrations at 7050 eV of the elements/compounds observed on the surface of the hard carbon electrodes extracted from a) half-cells (HC) and full-cells (FC) after the 1^st^, 2^nd^, and 10^th^ sodiation cycles. The plotted errors represent ± 2σ, where σ is the standard deviation in the fitted peak areas/atomic concentration calculated from the set of Monte Carlo simulations (refer to Note 1.2 in the SI).


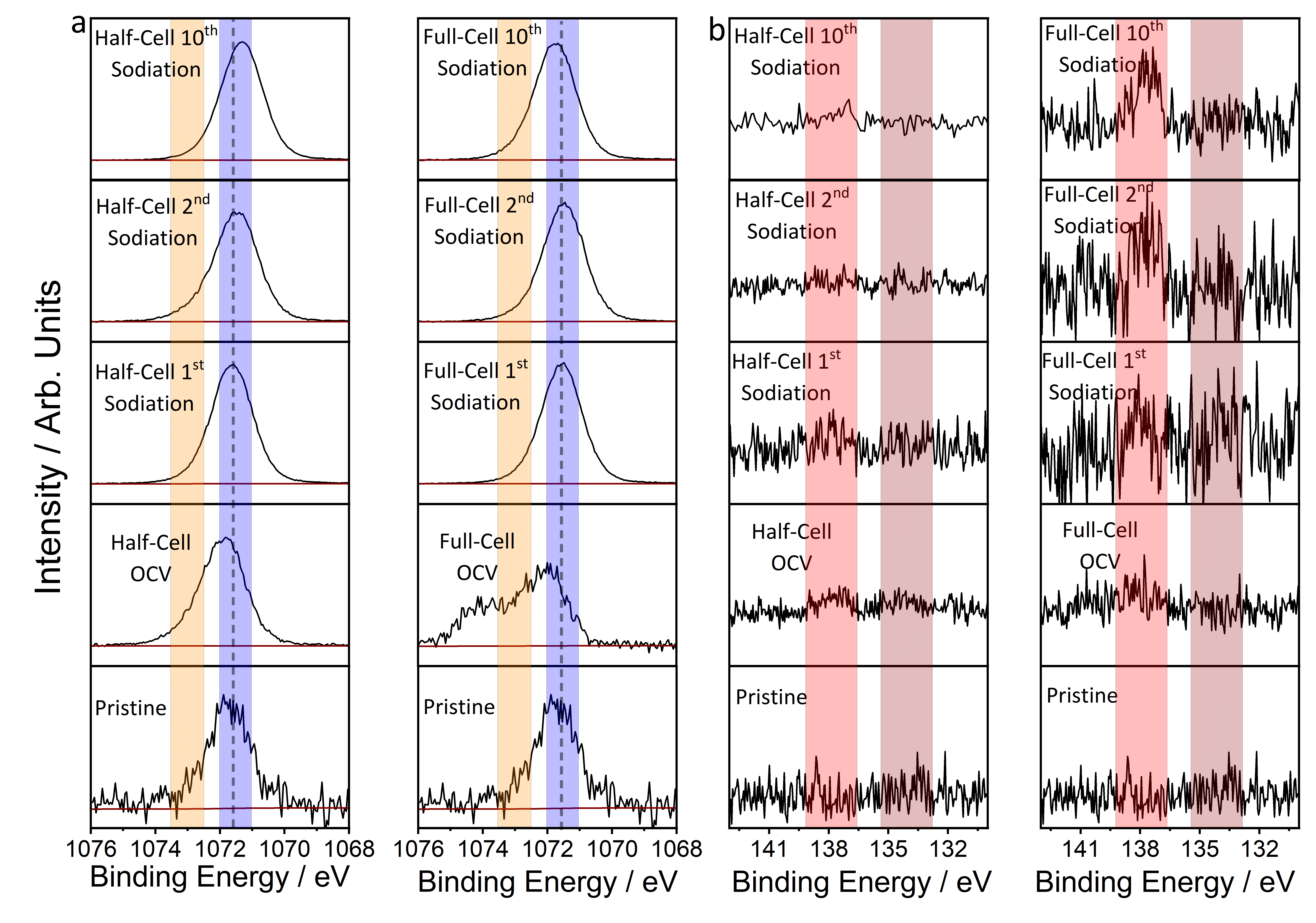

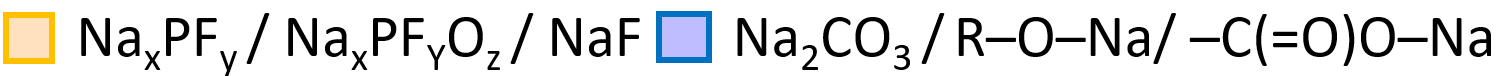

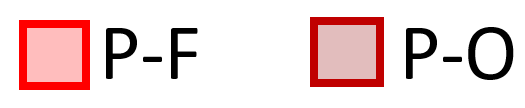


Figure S8. a) Na 1s and b) P 2p HAXPES spectra obtained at 7050 eV for hard carbon electrodes in the pristine state and extracted from half-cells and full-cells after a 10 h OCV rest period and the 1^st^, 2^nd^, and 10^th^ sodiation cycles. The dashed lines shown in a) are positioned at the center of the Na 1s peak for the half-cell and full-cell 1^st^ sodiation samples to emphasize the shift in the Na 1s peak as cycling progressed.


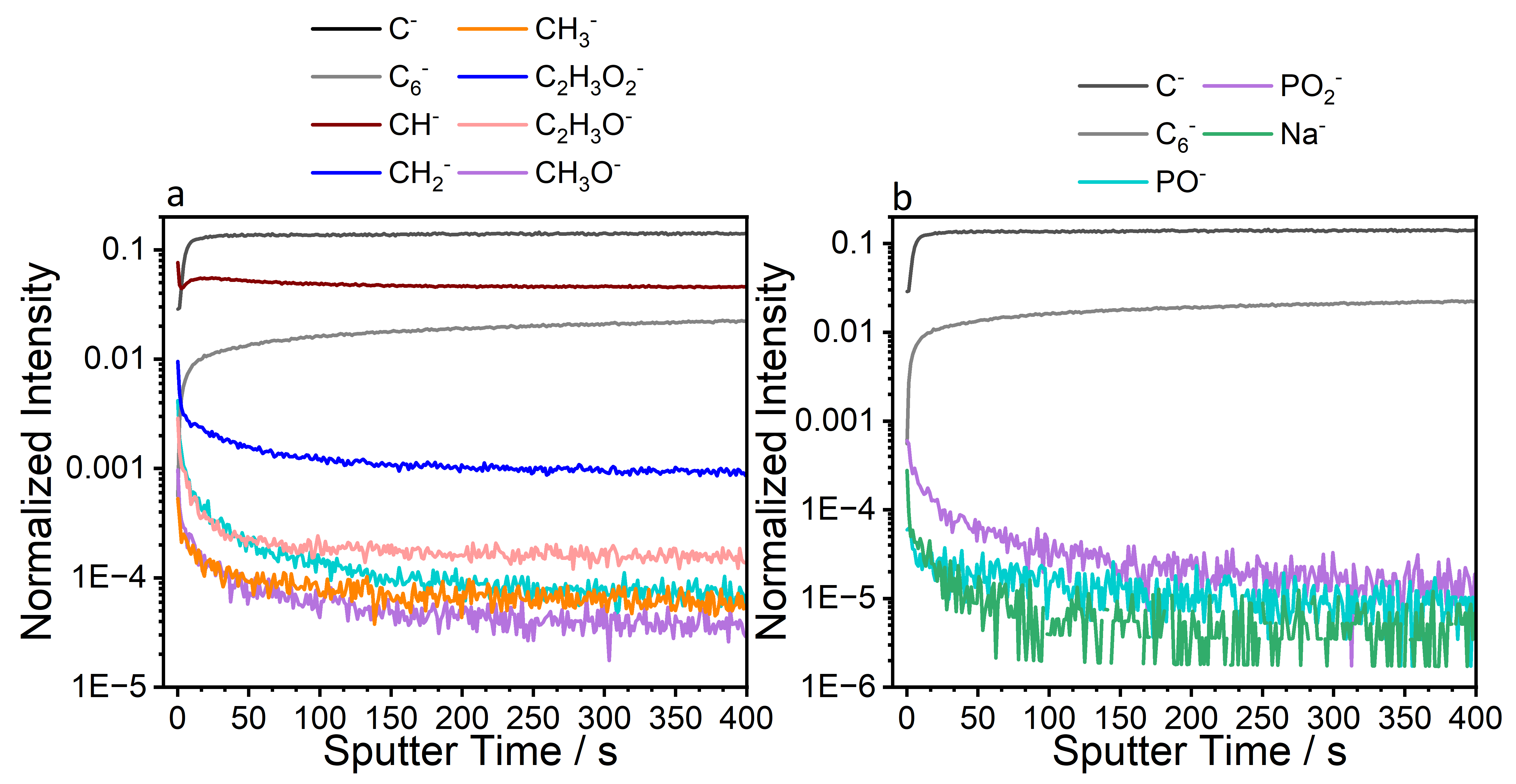
Figure S9. ToF-SIMS negative ion depth profiles of selected secondary ion fragments for the pristine hard carbon electrode. a) organic-containing fragments and b) inorganic-containing fragments.

# **References**

1. Cengiz, E. C., Rizell, J., Sadd, M., Matic, A. & Mozhzhukhina, N. Review—Reference Electrodes in Li-Ion and Next Generation Batteries: Correct Potential Assessment, Applications and Practices. *J Electrochem Soc* **168**, 120539 (2021).

2. Lee, S. E. & Tang, M. H. Reliable Reference Electrodes for Nonaqueous Sodium-Ion Batteries. *J Electrochem Soc* **166**, A3260–A3264 (2019).

3. Dugas, R., Forero-Saboya, J. D. & Ponrouch, A. Methods and Protocols for Reliable Electrochemical Testing in Post-Li Batteries (Na, K, Mg, and Ca). *Chemistry of Materials* **31**, 8613–8628 (2019).

4. El-Cell. PAT Cell Components. https://www.el-cell.com/products/cell-components/ (April 2025).

5. Kanamura, K., Okagawa, T. & Takehara, Z. ichiro. Electrochemical oxidation of propylene carbonate (containing various salts) on aluminium electrodes. *J Power Sources* **57**, 119–123 (1995).

6. Colbin, L. O. S. *et al.* Anodic dissolution of aluminum in non-aqueous electrolyte solutions for sodium-ion batteries. *Energy Advances* **3**, 143–148 (2023).

7. Ould, D. M. C. *et al.* New Route to Battery Grade NaPF6 for Na-Ion Batteries: Expanding the Accessible Concentration. *Angewandte Chemie International Edition* **60**, 24882–24887 (2021).

8. Liu, G. *et al.* Revisiting the sodium-ion storage capability of hard carbon in carbonate-based electrolytes via a sodium-metal-free protocol. *Sci China Chem* **67**, 2240–2247 (2024).

9. Dugas, R., Forero-Saboya, J. D. & Ponrouch, A. Methods and Protocols for Reliable Electrochemical Testing in Post-Li Batteries (Na, K, Mg, and Ca). *Chemistry of Materials* **31**, 8613–8628 (2019).

10. Iermakova, D. I., Dugas, R., Palacín, M. R. & Ponrouch, A. On the Comparative Stability of Li and Na Metal Anode Interfaces in Conventional Alkyl Carbonate Electrolytes. *J Electrochem Soc* **162**, A7060–A7066 (2015).

11. Müller, C. *et al.* Influences on Reliable Capacity Measurements of Hard Carbon in Highly Loaded Electrodes. *Batter Supercaps* **6**, e202300322 (2023).

12. Wang, H. *et al.* Internal pressure regulation enables reliable electrochemical performance evaluation of lithium-ion full coin cell. *J Power Sources* **600**, 234235 (2024).

13. Müller, V., Scurtu, R.-G., Memm, M., Danzer, M. A. & Wohlfahrt-Mehrens, M. Study of the influence of mechanical pressure on the performance and aging of Lithium-ion battery cells. *J Power Sources* **440**, 227148 (2019).

14. Biesinger, M. C. Accessing the robustness of adventitious carbon for charge referencing (correction) purposes in XPS analysis: Insights from a multi-user facility data review. *Appl Surf Sci* **597**, (2022).

15. Gengenbach, T. R., Major, G. H., Linford, M. R. & Easton, C. D. Practical guides for x-ray photoelectron spectroscopy (XPS): Interpreting the carbon 1s spectrum. *Journal of Vacuum Science & Technology A* **39**, 013204 (2021).

16. Major, G. H. *et al.* Practical guide for curve fitting in x-ray photoelectron spectroscopy. *Journal of Vacuum Science & Technology A* **38**, 061203 (2020).

17. NIST X-ray Photoelectron Spectroscopy Database, NIST Standard Reference Database Number 20. *National Institute of Standards and Technology, Gaithersburg MD* (2000)

18. Beamson, G. & Briggs, D. High resolution monochromated X-ray photoelectron spectroscopy of organic polymers: A comparison between solid state data for organic polymers and gas phase data for small molecules. *Mol Phys* **76**, 919–936 (1992).

19. Morgan, D. J. Comments on the XPS Analysis of Carbon Materials. *C (Basel)* **7**, 51 (2021).

20. Philippe, B. *et al.* Photoelectron Spectroscopy for Lithium Battery Interface Studies. *J Electrochem Soc* **163**, A178–A191 (2016).

21. Carboni, M. *et al.* Analysis of the Solid Electrolyte Interphase on Hard Carbon Electrodes in Sodium-Ion Batteries. *ChemElectroChem,* 1745–1753 (2019)

22. Alptekin, H. *et al.* Elucidation of the Solid Electrolyte Interphase Formation Mechanism in Micro-Mesoporous Hard-Carbon Anodes. *Adv Mater Interfaces* **9**, (2022).

23. Muñoz-Márquez, M. Á., Zarrabeitia, M., Passerini, S. & Rojo, T. Structure, Composition, Transport Properties, and Electrochemical Performance of the Electrode-Electrolyte Interphase in Non-Aqueous Na-Ion Batteries. *Advanced Materials Interfaces* **9**, 2101773 (2022)

24. Gond, R. *et al.* A Lignosulfonate Binder for Hard Carbon Anodes in Sodium-Ion Batteries: A Comparative Study. *ACS Sustain Chem Eng* **9**, 12708–12717 (2021).

25. Fondard, J. *et al.* SEI Composition on Hard Carbon in Na-Ion Batteries After Long Cycling: Influence of Salts (NaPF 6 , NaTFSI) and Additives (FEC, DMCF). *J Electrochem Soc* **167**, 070526 (2020).

26. Chen, L., Kishore, B., Walker, M., Dancer, C. E. J. & Kendrick, E. Nanozeolite ZSM-5 electrolyte additive for long life sodium-ion batteries. *Chemical Communications* **56**, 11609–11612 (2020).

27. Pan, Y. *et al.* Investigation of the solid electrolyte interphase on hard carbon electrode for sodium ion batteries. *Journal of Electroanalytical Chemistry* **799**, 181–186 (2017).

28. Pinder, J. W. *et al.* Avoiding common errors in X-ray photoelectron spectroscopy data collection and analysis, and properly reporting instrument parameters. *Applied Surface Science Advances* **19**, 100534 (2024).

29. Moeini, B. *et al.* Box plots: A simple graphical tool for visualizing overfitting in peak fitting as demonstrated with X-ray photoelectron spectroscopy data. *J Electron Spectros Relat Phenomena* **250**, 147094 (2021).
